# ChIP-seq profiling of H3K4me3 and H3K27me3 in an invasive insect, *Bactrocera* *dorsalis*

**DOI:** 10.3389/fgene.2023.1108104

**Published:** 2023-02-23

**Authors:** Yan Zhao, Juntao Hu, Jiajiao Wu, Zhihong Li

**Affiliations:** ^1^ Key Laboratory of Surveillance and Management for Plant Quarantine Pests, Ministry of Agriculture and Rural Affairs, College of Plant Protection, China Agricultural University, Beijing, China; ^2^ Ministry of Education Key Laboratory for Biodiversity Science and Ecological Engineering, Institute of Biodiversity Science, Center of Evolutionary Biology, School of Life Sciences, Fudan University, Shanghai, China; ^3^ Technology Center of Guangzhou Customs, Guangzhou, China

**Keywords:** epigenetics, histone modification, chromatin immunoprecipitation high throughput sequencing, Bactrocera dorsalis, flight activity

## Abstract

**Introduction:** While it has been suggested that histone modifications can facilitate animal responses to rapidly changing environments, few studies have profiled whole-genome histone modification patterns in invasive species, leaving the regulatory landscape of histone modifications in invasive species unclear.

**Methods:** Here, we screen genome-wide patterns of two important histone modifications, trimethylated Histone H3 Lysine 4 (H3K4me3) and trimethylated Histone H3 Lysine 27 (H3K27me3), in adult thorax muscles of a notorious invasive pest, the Oriental fruit fly *Bactrocera dorsalis* (Hendel) (Diptera: Tephritidae), using Chromatin Immunoprecipitation with high-throughput sequencing (ChIP-seq).

**Results:** We identified promoters featured by the occupancy of H3K4me3, H3K27me3 or bivalent histone modifications that were respectively annotated with unique genes key to muscle development and structure maintenance. In addition, we found H3K27me3 occupied the entire body of genes, where the average enrichment was almost constant. Transcriptomic analysis indicated that H3K4me3 is associated with active gene transcription, and H3K27me3 is mostly associated with transcriptional repression. Importantly, we identified genes and putative motifs modified by distinct histone modification patterns that may possibly regulate flight activity.

**Discussion:** These findings provide the first evidence of histone modification signature in *B. dorsalis*, and will be useful for future studies of epigenetic signature in other invasive insect species.

## Introduction

Understanding the mechanisms underlying animal responses to environmental change is essential to predict the shifts in animal distribution range (Parmesan, 2006; Merilä and Hendry, 2014; González-Tokman et al., 2020; Aguirre-Liguori et al., 2021). While a large number of studies have demonstrated the important role of genetic mechanism in animal responses to environmental stressors, such as temperature (Dixon et al., 2015; Cao et al., 2021; Chen and Narum, 2021), salinity (Berg et al., 2015; Garcia-Elfring et al., 2021; Rautsaw et al., 2021), oxygen (Yu et al., 2016; Yang et al., 2021; Zhang et al., 2021), and precipitation (Dudaniec et al., 2018; Gheyas et al., 2021; Wiener et al., 2021), a surge of recent evidence suggests that non-genetic mechanism, especially epigenetic modifications, can also contribute to responses to changing environment in animal populations with little genetic variation (Bossdorf et al., 2008; Richards and Pigliucci, 2020; Stajic and Jansen, 2021). Unlike genetic variation, epigenetic variation can directly interact with ambient environments, and induce possibly heritable gene expression changes without altering underlying gene sequence, thus providing a faster route for animals adapting to novel environments (Bossdorf et al., 2008; Feil and Fraga, 2012; Hu and Barrett, 2017). Epigenetic variation may therefore be an important mechanism underlying phenotypic variation and adaptive evolution within the context of limited genetic variation (Jaenisch and Bird, 2003; Verhoeven et al., 2016).

To date, the most extensively studied epigenetic mechanism is DNA methylation, which has been found involved in phenotypic responses to a number of environmental cues in animal populations [e.g., salinity (Artemov et al., 2017; Metzger and Schulte, 2018; Heckwolf et al., 2020), temperature (Marsh and Pasqualone, 2014; Paredes et al., 2016; Metzger and Schulte, 2017; Sheldon et al., 2020), photoperiod (Stevenson and Prendergast, 2013; Stevenson, 2018; Lindner et al., 2021), chemical pollutants (Baccarelli and Bollati, 2009; Devóz et al., 2017; Sanchez et al., 2017; Barouki et al., 2018)]. However, other epigenetic modifications underlying phenotypic plasticity have been less characterized in animals (Hu and Barrett, 2017; Jeremias et al., 2018; Vogt, 2021). The research bias toward DNA methylation also holds true in insect studies (Mukherjee et al., 2015; Maleszka, 2016; Glastad et al., 2019). With the exception for a few studies (Dai et al., 2018; Jones et al., 2018), almost all insect studies have focused on the relationship between DNA methylation variation and phenotypic change in model and non-invasive insects such as bees (Lyko et al., 2010; Foret et al., 2012; Yagound et al., 2020), ants (Bonasio et al., 2012; Libbrecht et al., 2016; Oldroyd and Yagound, 2021), aphids (Walsh et al., 2010; Mathers et al., 2019), and mealybug (Bain et al., 2021). Because it has been suggested that invasive species are more phenotypically plastic than non-invasive species in growth, morphological, and physiological traits (Davidson et al., 2011; Kelly, 2019; Bates et al., 2020), it is possible that the epigenetic patterns underlying plasticity are different between invasive and non-invasive insects. However, without proper profiling of regulatory landscape and functions, the role of epigenetic modifications other than DNA methylation in regulating phenotypes of invasive insects remains unclear.

In addition to DNA methylation, post-translational modifications of histones (hPTMs) including lysine acetylation, methylation, phosphorylation, sumoylation, and ubiquitinylation, are important epigenetic modifications of transcriptional regulation through altering chromatin structure (Kouzarides, 2007; Bonasio et al., 2010; Zhou et al., 2011). Genome-wide epigenomic profiles of specific histone modification and their transcriptional regulation roles have been previously reported in *Drosophila* (Kharchenko et al., 2011; Nègre et al., 2011). In particular, trimethylated Histone H3 Lysine 4 (H3K4me3) is usually found around the transcription start sites (TSSs) of genes, and targets active promoters (Bernstein et al., 2005; Mikkelsen et al., 2007; Benayoun et al., 2014), while trimethylation of Histone H3 Lysine 27 (H3K27me3) is usually located in intergenic regions, and acts as a repressive epigenetic marker to silence gene transcription (Grossniklaus and Paro, 2014; Aranda et al., 2015; Conway et al., 2015). In addition, the two marks can co-localize as bivalent domains to maintain gene expression levels at poised state (Bernstein et al., 2006; Vastenhouw and Schier, 2012). Studies of hPTMs in insects have been largely limited to the model species *Drosophila*, with hPTMs found involving in regulating phenotypic behaviors or changes such as circadian rhythm (Bu et al., 2020; Gong et al., 2021), aging (Siebold et al., 2010; Ma et al., 2018), and adaptation to stressful environments (Sharma et al., 2017; Wang et al., 2021). The few hPTMs studies in non-model insect species have focused on phenotypic change in non-invasive insects, such as nutritionally affected polyphenisms in honey bees and ants (Simola et al., 2013; Glastad et al., 2015; Simola et al., 2016; Wojciechowski et al., 2018), pupal diapause in response to photoperiod in the flesh fly and oil-collecting bee (Reynolds et al., 2016; Santos et al., 2018), and morphological development influenced by nutritional conditions in the flour beetle (Ozawa et al., 2016). Taken together, it is evident that hPTMs can obviously regulate important physiological processes and behavioral activities of insects. However, the role of histone modifications in invasive insects are still unknown.

**FIGURE 1:**
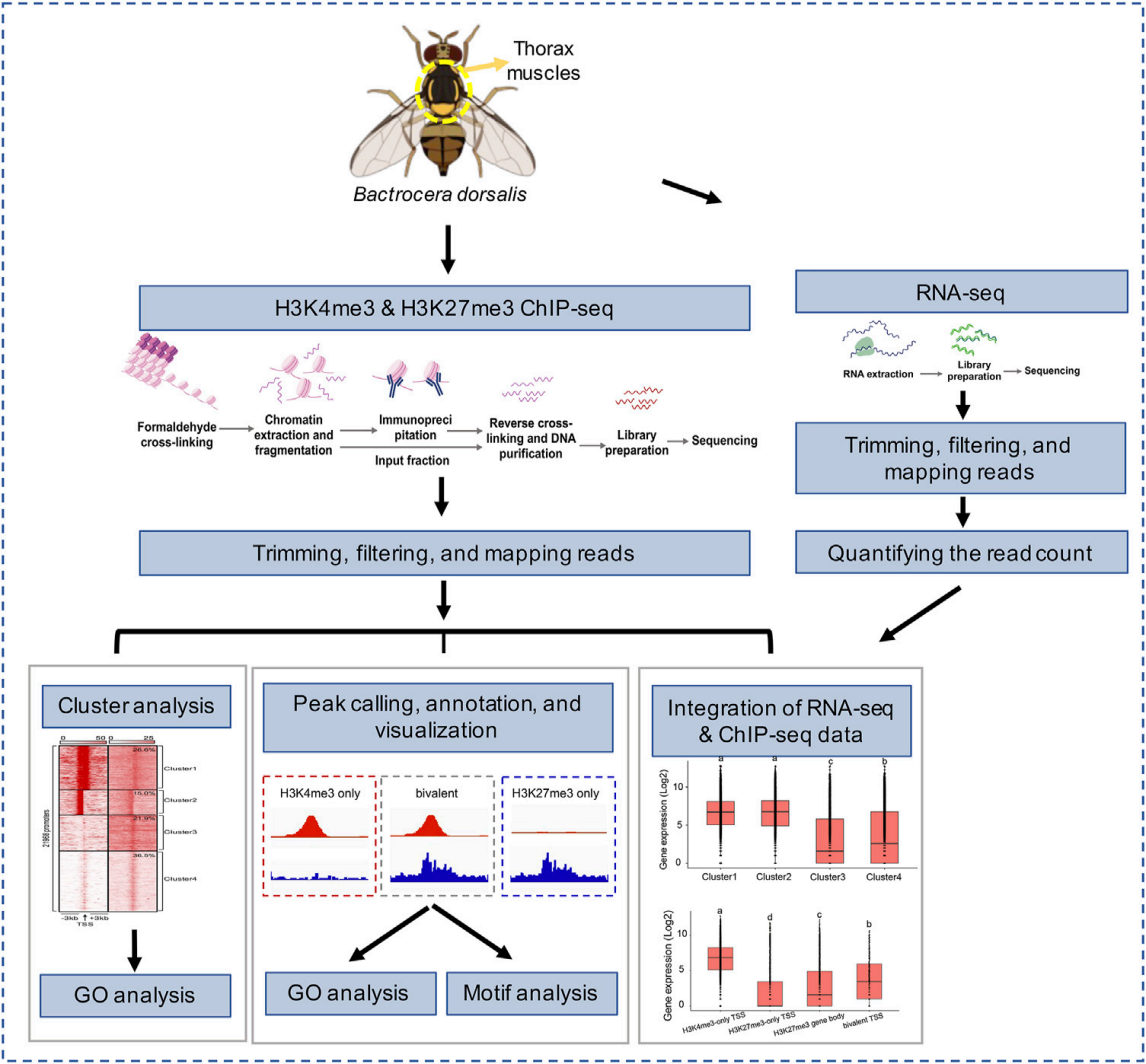
Overview of experimental design and data analysis.

To provide histone modification landscape of invasive insect species, we used *Bactrocera dorsalis* (Hendel) (Diptera: Tephritidae) (Figure 1), an notorious invasive species that has caused significant loss to global agriculture due to its polyphagia, high fecundity, superior mobility, high insecticide resistance, and strong environmental stress resistance (Santos-Rosa et al., 2002; Ekesi et al., 2007; Han et al., 2011; Jin et al., 2011; Hu et al., 2014; Magagula et al., 2015; Pieterse et al., 2017; Gu et al., 2019). After its first detection in 1912 in southern China, *Bactrcocera dorsalis* is now widely distributed in China with weak population genetic structure (Qin et al., 2018). Besides, it has strong flight capacity, and they can fly long distance or even fly to a high altitude and then disperse *via* moving air currents, which is prerequisite for long distance migration (Steiner, 1957; Chen et al., 2007). As its strong flight capacity is important for entry, establishment and spreading during invasiveness (Roques et al., 2009; Guo et al., 2018), it provides an excellent insect model to study the regulatory role of histone modification on insect flight activity. We used Chromatin Immunoprecipitation followed by high-throughput sequencing (ChIP-seq) (Furey, 2012; Park et al., 2017), which has been proven useful to identify transcription factor binding sites (Schmidt et al., 2010; Stefflova et al., 2013), conserved regulatory regions (Tena et al., 2014), and the activity of tissue-specific regulatory elements (Visel et al., 2009; Blow et al., 2010; Attanasio et al., 2013), to profile two histone modifications, H3K4me3 and H3K27me3, in thorax muscles, a key tissue to insect flight ability (Maughan and Vigoreaux, 1999; Josephson, 2006; Roques et al., 2009; Guo et al., 2018). We then correlated the histone modification profiles with levels of genome-wide gene expression obtained by RNA-seq, which allowed us to link enrichment or depletion of active and repressive histone modifications to their target genes. In this study we investigate two specific questions: a) What are the general regulatory landscapes of the two histone modifications in the flight muscles of this invasive insect? b) Is there functional relevance of the two histone modifications to flight activity? Answering the two questions will help us better understand the histone modification signature of the flight muscles of *B. dorsalis*, and serve as the baseline for future studies of mapping histone modification of other invasive insects.

## Materials and methods

### Insect source and husbandry

We collected *B. dorsalis* larvae individuals from the southern edge of its distribution in China (Danzhou, Hainan Province; 19°30′N, 108°50′E), within the initial entry region of this species in China, which could provide a baseline and more comprehensive genome-wide regulatory landscape of hPTMs in *B. dorsalis*. Then we reared the larvae for two generations in a common condition in lab as described in Li et al. (2012) to remove environmental effects from previous generations (Sanford and Kelly, 2011; Somero, 2012). Briefly, we distributed 500 eggs to the surface of an artificial diet (Supplementary Table S1) until larvae hatching. After hatching and emergence from pupae, we maintained all adult flies at a density of 200 individuals per rearing cage (45 × 45 × 50 cm), air temperature of 25°C ± 0.5°C, humidity of 70% ± 5%, and a photoperiod of 14 L: 10 D in an artificial climate cabinet (PXZ-430B, Ningbo Jiangnan Instrument Factory, China). We fed adult flies with 7.5 g sucrose and 2.5 g peptone (AOBOX, China) every 2 days. To control for developmental stage and sex, equal number of sexually matured, 15-day-old male and female adults were randomly selected, snap frozen in microcentrifuge tubes, and stored at −80°C until extraction of genetic material.

### Tissue of choice

The choice of tissue used for genome-wide mapping of histone modification can influence the interpretation of modification landscape (Bonn et al., 2012). Because the strong invasion ability of *B. dorsalis* is partially due to its remarkable flight capacity that significantly increases when reaching sexual maturity, we selected thorax muscles from sexually matured adults (Liang et al., 2001; Josephson, 2006), which is likely to increases the likelihood of identifying histone modification peaks and associated genes with functions relevant to flight activity.

### Chromatin immunoprecipitation

To identify the regulatory landscape of histone modification, we performed ChIP experiments using two independent biological replicates for both H3K4me3 and H3K27me3, with each replicate consisting of mixed 25 males and 25 females. We used two replicates by following the recommendation of ENCODE Consortium, because more than two replicates do not significantly improve site discovery (Rozowsky et al., 2009). To prepare ChIP libraries, we followed the protocol as described in Nagalingam et al. (2018) with some minor modifications. Briefly, we first dissected and pooled thorax muscle tissue (hereafter referred to as “muscles”) from 50 adults in cold 1X phosphate-buffered saline (PBS, P1022, Solarbio, China) mixed with 1X protease inhibitor (A8260, Solarbio, China). After thorough disruption and filtration through cell strainer, chromatin released from muscles was cross-linked in 200 μL of 1% fresh formaldehyde (HUSH, China) and cold PBS at room temperature for 15 min. We stopped the reaction by adding 1 M glycine (MG0167, MesGenBiotech, China) to a final concentration of 0.2M, and incubating in a rotator mixer at room temperature for an additional 10 min. The fixed tissues were washed three times using cold PBS, and homogenized by centrifuging at 4°C in a speed of 700*g* for 5 min. To lyse cells and retrieve single cell suspension, we resuspended pellets in 1 mL of animal cell nuclear lysate (20–163, Millipore, US) on ice for 30 min. The fragmentation of chromatin DNA was achieved by transferring 100 μL of the lysate into a sonication tube, and sonicating by Bioruptor^®^ UCD-200 to the length of 200–600 bp (Supplementary Figure S1A). After running electrophoresis on agarose gel to check sonication results, we diluted the supernatant containing sheared chromatin DNA with 900 μL ChIP dilution buffer (20–153, Millipore, US) containing 4.5 μL protease inhibitors, and mixed with 60 μL of Protein G Agarose (16–201D, Millipore, US) for 1 h at 4°C in a rotator mixer. After pellet agarose by brief centrifugation at a speed of 5000*g* for 1 min, we collected precleared chromatin from the remaining supernatant.

To set up ChIP reactions, we used anti-H3K4me3 (ab8580, Abcam) and anti-H3K27me3 (ab6002, Abcam) after searching antibodies from the Antibody Validation Database (http://compbio.med.harvard.edu/antibodies), and assessed their quality using Western blot. We performed Western blot following Marcon et al. (2015) with some minor modifications. Briefly, we first extracted histones, boiled them in sample buffer, ran stacking gel of 4.5% acrylamide in 10% polyacrylamide to electrophorese, and transferred the proteins from gel to PVDF membrane (Immobilon-P Transfer membrane, Millipore). Following incubation with primary and secondary antibodies, we performed chemiluminescence detection with Pierce ECL Western Blotting Substrate (Thermo Fisher Scientific, US) following the manufacturer’s instructions (Supplementary Figure S1B). We then separately mixed precleared chromatin with one of the two antibodies (5 ug of antibody per reaction) following manufacture’s instruction, and incubated the mix overnight on a rotator mixer at 4°C. We added 60 μL Protein G Agarose to each reaction, and incubated all reactions on a rotator mixer at 4°C for 1 h to collect the antibody/antigen/DNA complex. Beads were then sequentially washed once with Low Salt Immune Complex Wash Buffer (20–154, Millipore, US), High Salt Immune Complex Wash Buffer (20–155, Millipore, US), LiCl Immune Complex Wash Buffer (20–156, Millipore, US), and twice with TE Buffer (20–157, Millipore, US). DNA was then eluted from the beads by incubation in 200 μL elution buffer (10 μL 20% SDS, 20 μL 1 M NaHCO3 and 170 μL ddH_2_O) for 15 min at room temperature. We then added 8 μL 5 M NaCl to all samples, followed by incubation at 65°C overnight to reverse the DNA-protein crosslinks. Then, we incubated the samples for 30 min at 37°C with 1 μL RNase A (R6513, Merck, Germany), followed by mixing with 4 μL 0.5 M EDTA (E8030, Solarbio, China), 8 μL 1 M Tris-HCl (T1020, Solarbio, China) and 1 μL proteinase K (4333793, ThermoFisher, US) and incubating at 45°C for 2 h. Finally, DNA was purified using Bioline DNeasy minikit following manufacturer’s protocol. The final concentration and amount of DNA used for ChIP-seq library preparation were shown in Supplementary Figure S1C.

### Library preparation and sequencing

ChIP-seq libraries were prepared, and sequenced by following the ENCODE guidelines (Landt et al., 2012) at Wuhan IGENEBOOK Biotechnology Co., Ltd. DNA fragments ranging from 200 to 300 bp were selected using SPRI beads, and amplified by 15 PCR cycles following end repairing and adapter ligation. The quality of libraries was checked on Bioanalyzer 2100 (Agilent) and Qubit fluorometer (Invitrogen, Carlsbad, CA, United States). All ChIP-seq libraries were sequenced in one lane (150-bp pair-end reads) of Illumina HiSeq 2000 platform.

### Quality control and read mapping

To remove adapter contamination, low-quality bases, and bases artificially introduced during library construction, we filtered ChIP-seq reads following the steps described in Zhou et al. (2011) with some modifications. We examined read quality of each sample using FASTQC v0.11.8 (http://www.bioinformatics.babraham.ac.uk/projects/fastqc/), and trimmed reads using Trim Galore! v0.6.6 (www.bioinformatics.babraham.ac.uk/projects/trim_galore/) with default parameters. We used Bowtie2 v2.4.2 (Langmead and Salzberg, 2012) to align trimmed reads for each replicate to the genome (NCBI assembly accession ASM78921v2) with default parameters. We only included reads that uniquely mapped to the reference genome in downstream analysis. We used SAMtools v1.3.1 (Li et al., 2009) to sort mapped reads based on reference coordinates, and filtered unmapped, non-uniquely mapped and discordantly paired reads by setting ‘view -F 3852’ and ‘view -b -f 2’, and removed reads with low mapping quality (MAPQ <30). PCR duplicated reads were removed by using MarkDuplicates function in Picard v2.25.1 (http://broadinstitute.github.io/picard/). We checked the reproducibility between biological replicates by calculating the Pearson correlation coefficient (PCC) and Spearman’s correlation coefficient (SCC) for genome-wide read distribution, using the average signals in individual 500-bp non-overlapping genomic windows with “multiBamSummary” and “plotCorrelation” functions in deepTools v3.5.1 (Bardet et al., 2012; Ramirez et al., 2014). We used Integrative Genome Viewer (IGV) v2.9.4 (Thorvaldsdottir et al., 2013) for visualization of enrichment profiles. Read counts were normalized to counts per million mapped reads (CPM) in 10-bp windows with the extendReads to 200, and converted to bigWig format using the “bamCoverage” function in deepTools.

### Evaluating ChIP-seq data

We used a number of indicators to evaluate the quality of the ChIP-seq data, following the Encyclopedia of DNA elements Consortium (ENCODE) guideline (Robertson et al., 2007; Landt et al., 2012). We considered a library to be highly complex when its non-redundant fraction (NRF) was at least 0.8, PCR Bottlenecking Coefficients (PBC) 1 was higher than 0.5 and PBC2 was higher than 1. In addition to peak calling, we separately quantified enrichment by conducting a strand cross-correlation analysis to assess the degree of immunoprecipitated fragment clustering in ChIP-seq experiments. We considered a library with good clustering when its normalized strand coefficient (NSC) was higher than 1.05, and its relative strand correlation (RSC) was higher than 0.8. Finally, we used fraction of reads in peaks (FRiP), which evaluates the number and strength of peaks obtained for each ChIP replicate, to measure the signal-to-noise ratio (S/N). We considered an immunoprecipitation reaction successful when its FRiP was at least 0.01.

### Cluster analysis

To explore the enrichment of aligned ChIP-seq reads for H3K4me3 and H3K27me3 in the 3-kb windows around the transcription starts sites (TSSs) (hereafter referred to as ‘TSS regions’), we first extracted reads and reference coordinates (position of 21,968 TSSs, assumed to be the 5′-end of all annotated transcripts), using the ‘getPromoter’ function implemented in the R package ChIPseeker v1.26.2 (Yu et al., 2015). Windows of negative strand transcripts were inverted so that they have the same orientation as the positive strand transcripts. Following Kratochwil and Meyer (2015), we performed clustering normalization using *K*-means linear, and distinguished between four categories of clusters: 1) Cluster 1, which are clusters featured by dual peaks of H3K4me3 around TSSs, with one peak highly occupied (mean density ≥200) and shifted to the 3′-end of TSSs (<200bp) and the other peak moderately occupied (20 ≤ mean density <200) and shifted to the 5’ -end of TSSs (<400bp), and very low or no (0 ≤ mean density <10) H3K27me3 occupancy around TSSs, 2) Cluster 2, which are clusters featured by one H3K4me3 peak highly occupied and shifted to the 5′ end of TSSs (<200 bp), and very low or no H3K27me3 occupancy around TSSs, 3) Cluster 3, which are clusters featured by low (10 ≤ mean density <20) H3K4me3 and H3K27me3 occupancy around TSSs, and 4) Cluster 4, which are clusters of featured by one H3K4me3 peak of low occupancy and very low or no H3K27me3 occupancy around TSSs. Finally, we annotated genes with their TSSs within the 3 kb distance to Cluster 1-3, using the ‘annotatePeak’ function implemented in the ChIPseeker for functional analysis. We visualized genomic distribution of clusters in heatmaps, using Seqminer v1.3.4 (Ye et al., 2011).

### Peak calling and genomic context analysis

To perform peak calling for each replicate, we used ‘ChIP-seq’ function, implemented in MACS2 v2.1.1 (Feng et al., 2012), with input group as control. For narrow peak such as H3K4me3, we determined reproducible peaks between replicates using irreproducible discovery rate (IDR) (Babu et al., 2011; Li et al., 2011). We used the ‘callpeak’ module with following parameters: -g 4.53e8 --keep-dup all, and a narrow peak cut-off of *p*-value <0.01. We then used the ‘idr’ module to measure the reproducibility of replicates, and ranked the peaks by their *p*-values with an IDR threshold of 0.01. Following the ENCODE ChIP-seq guidelines (Landt et al., 2012), peaks that passed the IDR threshold by comparing true replicates were retained for downstream analysis. For broad peak such as H3K27me3, we identified peaks by using the ‘callpeak’ module with following parameters: ‘--broad -g 4.53e8 --broad-cutoff 0.1 --keep-dup all’. We then identified the overlapping peaks between both replicates using the ‘findOverlapsOfPeaks’ function in the R package ChIPpeakAnno v3.10.2 (Zhu et al., 2010; Zhu, 2013). We used BEDtools v2.30.0 (Quinlan and Hall, 2010) intersect commands to identify the bivalent domains, which consist of nucleosomes containing both H3K4me3 and H3K27me3 simultaneously, with at least a 100-bp overlap for the estimation of one nucleosome overlap (Ku et al., 2021). We then mapped the H3K4me3 and H3K27me3 peaks to TSS and gene body regions [defined as the genomic region from the TSS to the transcription termination site (TTS) (Young et al., 2011)], respectively, to generate tagMatrix using the ‘getTagMatrix’ function in ChIPseeker, and visualized average profiles of the peaks binding to TSS regions (i.e., 5′ to 3′) and gene body regions, respectively.

To identify the positions of H3K4me3 and H3K27me3 peaks within genomic features, we annotated identified peaks to the *B. dorsalis* genome, using ‘annotatePeak’ function in ChIPseeker with default parameters. We gave the precedence to promoter >5′untranslated region (UTR) > 3′UTR > exon > intron > downstream > intergenic regions when features overlapped, and defined downstream regions as downstream 300 bp from the gene end. We then compared the distributions of peaks to null distributions of individual genomic features in the reference genome using *G* test.

### Effect on histone modification on gene expression

To demonstrate the regulatory role of the two histone modifications in gene expression, we constructed RNA-seq libraries for the same thorax muscle tissues as described above. We extracted total RNA using TRIzol reagent kit (15596–018, Invitrogen, United States) following the manufacturer’s protocol. The quality and concentration of extracted RNA was evaluated using Nanodrop 8000 spectrophotometer (Thermo Fisher, United States) and Agilent 2100 Bioanalyzer (Agilent Technologies, United States). RNA-seq libraries were prepared by Novogene Bioinformatics Technology Co. Ltd. (Tianjin, China) using NEBNext^®^ UltraTM Directional RNA Library Prep Kit (E7420, NEB, United States), and sequenced on the Illumina NovaSeq 6000 platform (Novogene) with 150-bp paired-end reads.

To quantify gene expression, we used Trim Galore! to remove adapter sequences and low-quality reads. The remaining quality-filtered reads were assessed with FASTQC and mapped to the *B. dorsalis* genome (NCBI Assembly accession ASM78921v2) using HISAT2 v2.1.0 (Kim et al., 2015), with the following parameters: --new-summary -k 1 --rna-strandness RF. We removed PCR duplicated reads using the MarkDuplicates function implemented in Picard. We used HTSeq-Count v0.12.4 (Anders et al., 2015) to output read counts for each gene with parameters “-f bam -s reverse -r name -t exon -union”. Finally, we compared the average expression levels (log2 of RPKM values) among genes 1) associated with Cluster 1–4, 2) with their TSSs enriched by H3K4me3, H3K27me3 or bivalent domains, and 3) with their gene bodies enriched by H3K27me3 using one-way analysis of variance (ANOVA).

### Gene annotation and gene ontology analysis

To perform the functional analysis of peaks, we identified genes with TSS regions enriched by H3K4me3, H3K27me3, or both peaks, using the ‘annotatePeak’ function in ChIPseeker. In addition, because H3K27me3 is also featured with broad domains across gene bodies, we thus also annotated genes with their gene bodies enriched with H3K27me3 peaks.

For gene ontology (GO) enrichment analysis, we first complied a GO term list for all protein-coding genes in the *B. dorsalis* genome, using eggNOG-mapper (Huerta-Cepas et al., 2017) to search for orthologous genes in the eggNOG database. The list was then used as input to the universal enrichment protocol, using the R package clusterProfiler v4.0.5 (Yu et al., 2012) and *q*-value cut-off of 0.001 to determine enrichment significance.

### Motif analysis

Because it has been suggested that motif analysis can facilitate the discovery of unanticipated sequence signals such as transcription factor binding sites associated with histone modifications (Bailey et al., 2013; Ruiz et al., 2019; Chen et al., 2020), we performed *de novo* motif analysis for H3K4me3, H3K27me3 and bivalent domains. We first extracted a list of sequences that are 50 bp upstream and 50 bp downstream from the summits of the top 500 peaks overlapping with TSS regions (Niu et al., 2018). We then used the list as input to search for conserved sequence motifs, using motif elicitation (MEME)-ChIP (Bailey et al., 2009) software with default parameters. We used TOMTOM (Khan et al., 2018) with default settings to match discovered motifs to the JASPAR database (Fornes et al., 2020). Finally, we used FIMO (Hertz and Stormo, 1999; Grant et al., 2011) to map motif prediction, and visualize genomic locations of the motifs in gene list as described above.

### Potential regulatory role of histone modifications in flight activity

As flight activity is closely related to wing development, and long-distance migration is an important and widespread flight activity, we acquired a list of previously documented genes involved in wing development of *B. dorsalis* and migration-associated genes from recent papers (Supplementary Table S2), and compared them to peak-annotated genes identified above to investigate the possible functional relevance of histone modification to flight activity (Jones et al., 2015; Guo et al., 2018; Doyle et al., 2022). Specifically, Guo et al. (2018) profiled transcriptomes of *B. dorsalis*, and identified a group of key genes with functions relevant to wing development, which showed highest expression level in the pupal stage and poised state in other stages, through comparative transcriptome analyses. Jones et al. (2015) used comparative transcriptomics of flight phenotypes to determine a suite of expressed candidate genes associated with flight activity in the cotton bollworm, *Helicoverpa armigera*, including odorant binding proteins, flight muscle structure, fatty acid synthesis, etc. Doyle et al. (2022) undertook a genome-wide transcriptomic comparison of actively migrating marmalade hoverfly, *Episyrphus balteatus* and found the features of the migrant phenotype have arisen by the integration and modification of pathways such as insulin signalling for diapause and longevity, JAK/SAT for immunity, and those leading to octopamine production and fuelling to boost flight capabilities. Specifically, upregulated genes associated with migration include genes related to metabolic processes, sensory functions, octopamine synthesis, neuropeptide hormones, muscle function, and immunity genes, and the downregulated genes include genes involved in insulin and TGF-β signalling, hormonal regulation, and *multiple ankyrin repeats single KH domain* (*mask*). We then compared the proportion of peak-annotated genes in each gene category to the proportion of peak-annotated genes in the reference genome using G test.

## Results

### Mapping statistics and quality check of ChIP-seq data in *B. dorsalis*

After alignment, 55.4%–55.7% of H3K4me3 reads, 40.2%–40.3% of H3K27me3 reads, and 37.3% of input reads were unambiguously mapped to the reference genome, with high PCC and SCC values between replicates, suggesting high experimental reproducibility (Supplementary Table S3; Supplementary Figure S3A). While 2.2% of H3K4me3 reads, 2.4%–2.6% of H3K27me3 reads, and 3.4% of input reads were filtered due to nonspecific matches, 42.1%–42.4% of H3K4me3 reads, 57.2%–57.3% of H3K27me3 reads, and 59.3% of input reads did not map at all. After further filtering of PCR duplicates, unmapped, non-uniquely mapped, improperly paired, and low mapping quality reads, 36.1%–36.3% of H3K4me3 reads, 17.4%–17.8% of H3K27me3 reads, and 19.6% of input reads were retained for downstream analysis (Supplementary Figure S2).

When analyzing uniquely mapped reads, all replicate and input samples passed the PBC1 and PBC2 quality threshold, while four of five samples failed to meet the NRF cut-offs (Supplementary Figure S4A), suggesting low library complexity, similar to the recent studies in other non-model species (Kingsley et al., 2019). However, when checking the NSC and RSC values, all samples met the ENCODE standard, and had larger fragment-length peaks compared to read-length peaks (Supplementary Figure S4B), suggesting high immunoenrichment quality despite lower library complexity. When checking FRiP value, both H3K4me3 replicate samples produced a high proportion of aligned reads within peaks, with the FRiP scores ranging from 0.611 to 0.612 (Supplementary Figure S4C), and the called regions ranging from 7,695 to 7,814 (Supplementary Figure S4D). In contrast, both H3K27me3 replicate sample had lower FRiP value of 0.295–0.319 and 14,929–16,481 called regions (Supplementary Figures S4C, S4D). In summary, we found that ChIP-seq can generally be adapted to the non-model invasive species *B. dorsalis*, and provide sufficient data for regulatory landscape analysis.

### Cluster analysis

**FIGURE 2:**
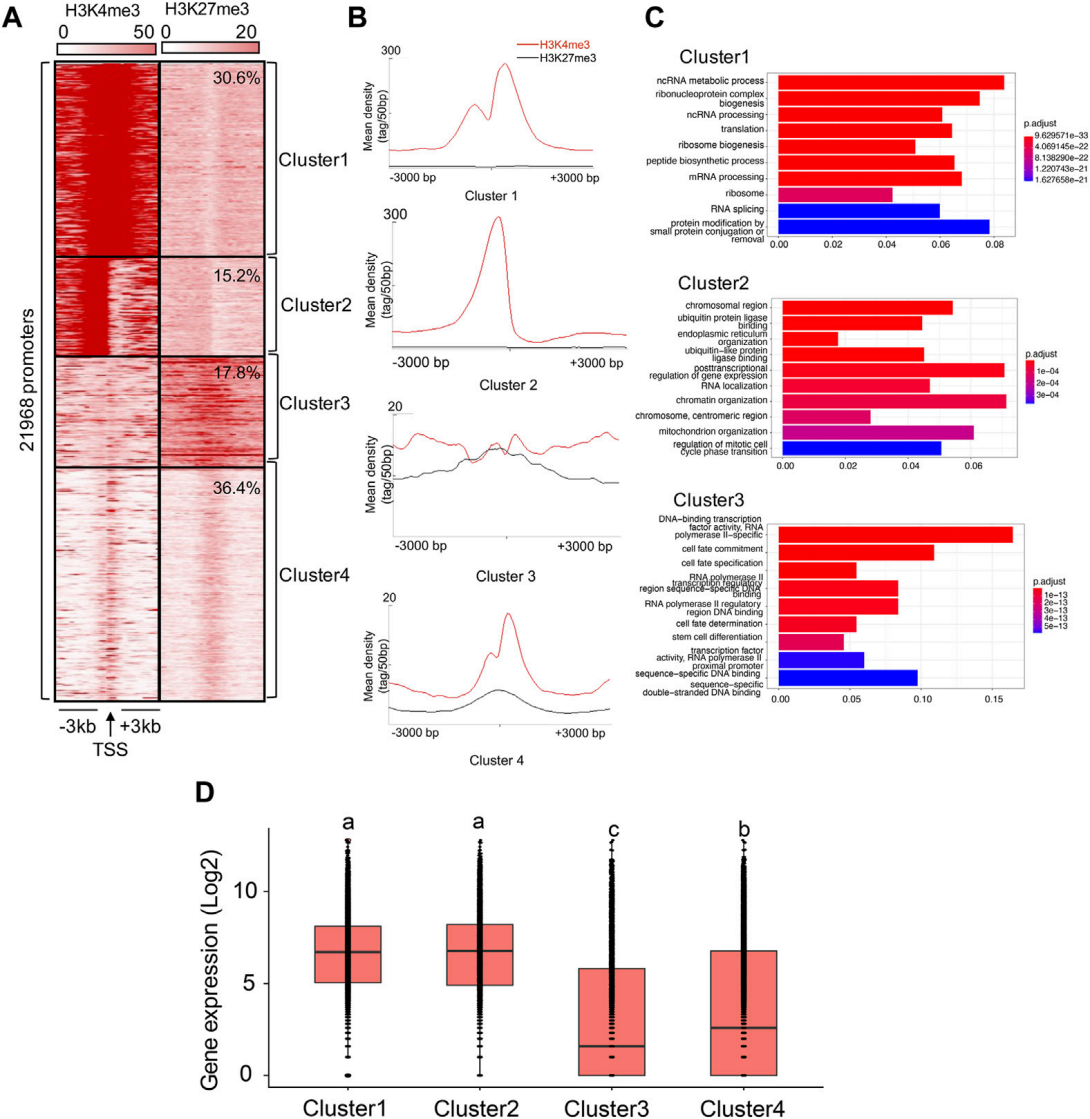
Cluster analysis of H3K4me3 and H3K27me3 in the *B. dorsalis* genome. **(A)** Heatmap and **(B)** density profile of the *K*-means clustering of mapped read position for H3K4me3 and H3K27me3 around TSS regions (±3 kb) in the *B. dorsalis* genome. **(C)** Top 10 over-represented GO terms of genes associated with Cluster 1–3. Corrected *p*-values are indicated by color, the warmer the color, the smaller the *p*-values. The vertical axis represents the GO term names and the horizontal axis represents gene ratio. **(D)** Boxplots showing the mean level of gene expression for genes with the TSSs within the 3 kb distance to Cluster 1–4. The gene expression levels were calculated based on the log2 of RPKM values. The differences in gene expression levels among clusters were evaluated using one-way analysis of variance (ANOVA).

We present the results obtained by running the analysis on Replicate 1 (H3K4me3_1 and H3K27me3_1) here. Results concerning Replicate 2 (H3K4me3_2 and H3K27me3_2) were similar (Supplementary Figure S6). Based on *K*-means cluster analysis, we found 30.6%, 15.2%, 17.8%, and 36.4% of TSSs falling into Cluster 1-4, respectively (Figures 2A, B). Therefore, we assumed that 45.8% of the genes were in either active or poised (Cluster 1 and Cluster 2) state, while 54.2% of the genes were in either weakly expressed (Cluster 3) or inactive (Cluster 4) state. Indeed, the gene expression patterns involved in each cluster have supported it (Cluster 1: 6.4 ± 0.02, Cluster 2: 6.4 ± 0.03, Cluster 3: 3.0 ± 0.04, Cluster 4: 3.6 ± 0.03; the gene expression were calculated based on the log2 of RPKM values), with the average expression levels of genes associated with Cluster 1 and Cluster 2 were significantly higher than those in Cluster 3 and Cluster 4 (ANOVA test, *p* < 0.05, Figure 2D).

### H3K4me3 and H3K27me3 peak analyses

**FIGURE 3:**
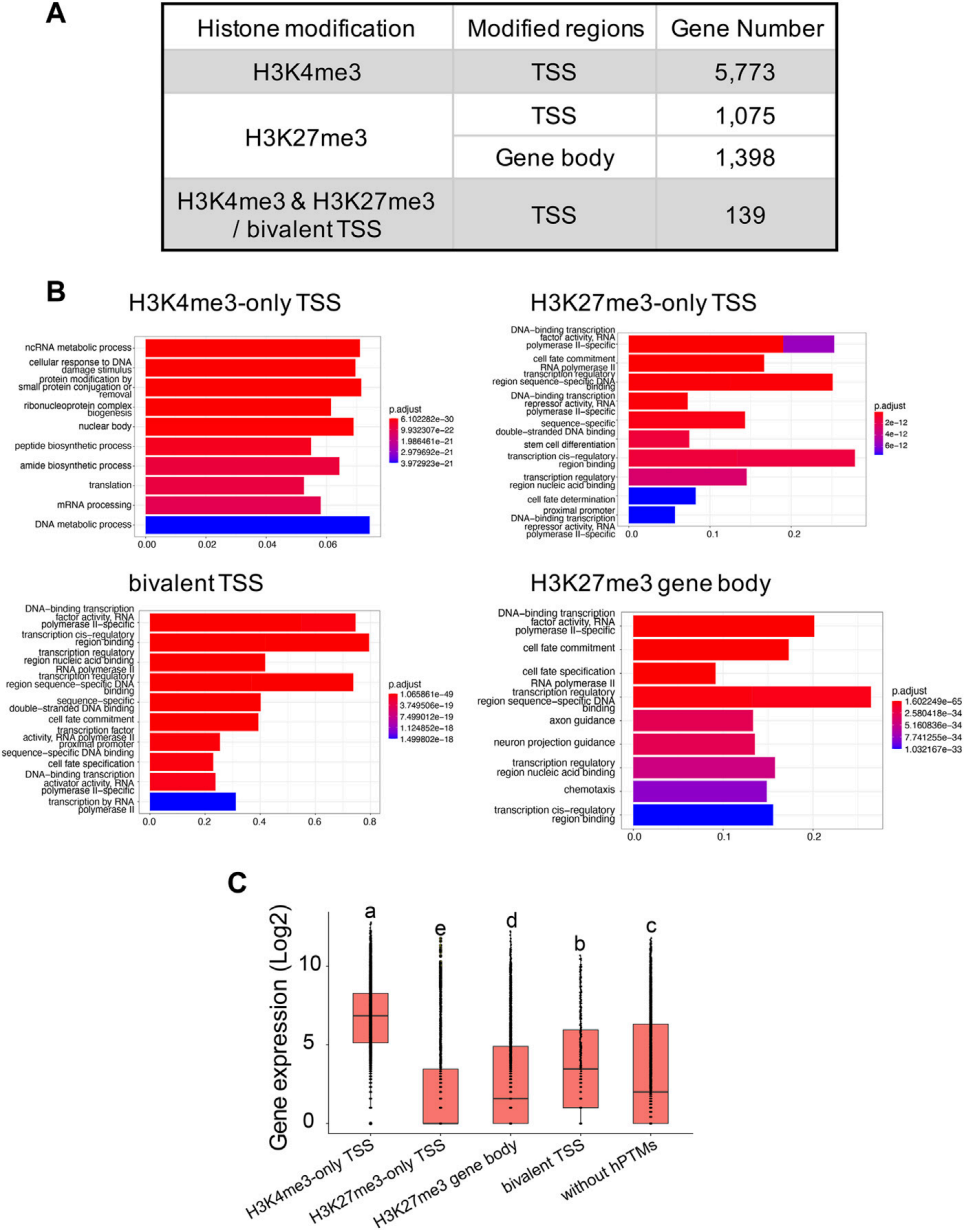
H3K4me3 and H3K27me3 peak analyses in the *B.* genome. **(A)** Summary of the genes with H3K4me3-only, H3K27me3-only and bivalent domains at the TSSs, and genes with H3K27me3 at the gene body regions in *B. dorsalis*. **(B)** Top 10 over-represented GO terms in genes with H3K4me3-only, H3K27me3-only and bivalent domains at the TSSs, and genes with H3K27me3 at the gene body regions in *B. dorsalis*. Corrected *p*-values are indicated by color, the warmer the color, the smaller the *p*-values. The vertical axis represents the GO term names and the horizontal axis represents gene ratio. **(C)** Boxplots showing the mean level of gene expression for genes with TSSs enriched by H3K4me3, H3K27me3, or both peaks and genes with gene bodies enriched by H3K27me3 and genes that are not modified by hPTMs. The gene expression levels were calculated based on the log2 of RPKM values. The differences in gene expression levels among different modifications were evaluated using one-way analysis of variance (ANOVA).

In total, we identified 7,226 and 11,417 peaks for H3K4me3 and H3K27me3, respectively (Supplementary Table S4), with the two replicates displaying high reproducibility and low noise interfering (Supplementary Figures S3, S5). 95.0% (6,865 out of 7,226) of H3K4me3 peaks and 45.9% (5,206 out of 11,417) of H3K27me3 peaks overlapped with 45.6% and 11.2% of genes annotated in *B. dorsalis* genome, respectively, suggesting that these two histone marks were widely involved in regulatory activities in this species. When analyzing the length of peaks, 45.3% of the H3K4me3 peaks had a length shorter than 1 kb, and 44.9% of peaks had a length ranging between 1 kb and 2 kb. As for H3K27me3 peaks, the length ranged from 251bp to 47,936 bp, with the majority of peaks (51.7%) having length longer than 1 kb. We extracted 5,541 and 843 genes with TSS regions annotated with H3K4me3-only and H3K27me3-only peaks, respectively, and we also obtained 295 bivalent domains, in which 197 bivalent domains were overlapped with 139 gene TSS regions (Figure 3A; Supplementary Table S5). Due to the key regulatory role of H3K27me3 locating within gene bodies, we also identified 1,398 genes with their gene bodies overlapped with H3K27me3 peaks. We then assessed the relationship between different histone modification profiles and gene expression. We found significant differences in average gene expression levels between H3K4me3-only TSS (log2 of RPKM values: 6.6 ± 0.02), H3K27me3-only TSS (2.1 ± 0.06), H3K27me3 gene body (2.7 ± 0.05), bivalent TSS (3.8 ± 0.2), and genes without hPTMs (3.3 ± 0.04), with the highest average expression levels were H3K4me3-only TSS, followed by bivalent TSS, genes without hPTMs and H3K27me3 gene body, and the lowest average expression levels were H3K27me3-only TSS (ANOVA test, *p* < 0.05, Figure 3C). However, genes with TSSs enriched by bivalent domains were not in the posited state, defined by Gaertner et al. (2012) as those with transcript levels below 10 RPKM as determined by RNA-seq, which was consistent with that in *Drosophila melanogaster*.

**FIGURE 4:**
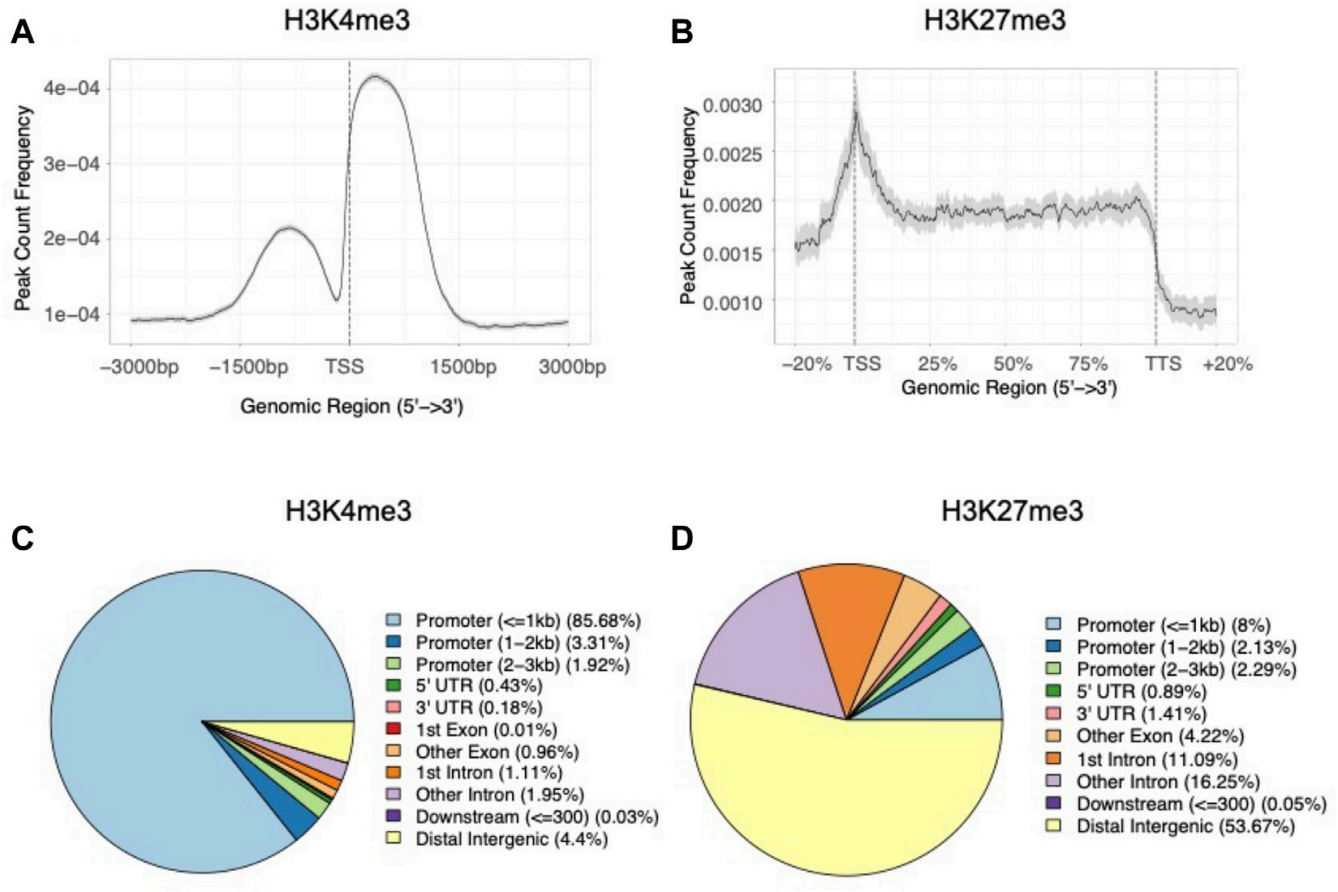
Distribution patterns of **(A)** average H3K4me3 peaks around TSS regions (±3 kb) and **(B)** average H3K27me3 peaks in gene body regions of the *B. dorsalis* genome. The proportion of genomic features of **(C)** H3K4me3 peaks and **(D)** H3K27me3 peaks compared with genomic features.

When examining the average histone modification peak profiles, we found a mutually exclusive pattern between the two marks where H3K4me3 peaks showed a strong dual peak pattern with a central notch around the TSSs (Figure 4A). In contrast, H3K27me3 tended to occupy broader regions, with the average enrichment was almost constant throughout gene bodies (Figure 4B). When analyzing the genomic features of H3K4me3 peaks, we found a significant enrichment of peaks in genic regions (*G* = 38.5, df = 1, *p* = 5.4 × 10^−10^), and promoter regions (within 1 kb upstream and 1 kb downstream to TSSs) (*G* = 419.4, df = 1, *p* < 2.2 × 10^−16^) when compared to the null distribution built on genome GFF annotation file (Figure 4C; Supplementary Table S5). While as for H3K27me3 peaks, only 8.0% of the peaks overlapped with the promoter regions (within 1 kb upstream and 1 kb downstream to TSSs) (*G* = 1.2, df = 1, *p* = 0.3) and more than half of the peaks located within intergenic regions (*G* = 28.3, df = 1, *p* = 1.0 × 10^−7^) (Figure 4D; Supplementary Table S5). In summary, the peak annotation results showed contrasting distribution patterns between H3K4me3 and H3K27me3, suggesting possibly distinct regulatory roles between the two modifications (Benayoun et al., 2014; Aranda et al., 2015; Conway et al., 2015).

### Functional analysis of clusters and peaks

We found a number of GO terms significantly enriched within each Cluster (Figure 2C; Supplementary Table S6), with genes involved in metabolic, biosynthetic processes, cell cycle, gene expression and chromatin organization significantly overrepresented (*q*-value <0.001) in Cluster 1 (503 GO terms) and Cluster 2 (37 GO terms). In contrast, genes annotated with Cluster 3 (229 GO terms) displayed different GO term enrichment, with functions related to cell fate, tissue development and morphogenesis, negative regulation of transcription by RNA polymerase II, and imaginal disc development significantly enriched (*q*-value <0.001). Genes associated to H3K4me3 vs. H3K27me3 also differed in the number and functions of GO terms (Figure 3B; Supplementary Table S6). Genes with TSSs uniquely modified by H3K4me3 significantly enriched in 622 GO terms (*q*-value <0.001) related to GO:0034660 (ncRNA metabolic process), GO:0006974 (cellular response to DNA damage stimulus), and GO:0006412 (translation), which were totally different from the GO terms associated with genes with TSSs uniquely modified by H3K27me3, which were enriched for GO:0001227 (DNA-binding transcription repressor activity, RNA polymerase II-specific), GO:0000976 (transcription cis-regulatory region binding) and GO:0021953 (central nervous system neuron differentiation). In contrast, we found 159 GO terms shared between the genes with TSSs (161 GO terms) and gene bodies (735 GO terms) modified by H3K27me3. Genes with bivalent protomers significantly enriched in 381 GO terms related to cell fate, transcription cis-regulatory, tissue development and morphogenesis, and imaginal disc development, with 104 GO terms overlapped with GO terms associated with genes uniquely modified by H3K27me3 at TSS regions and gene bodies, and only two GO terms, GO:0003712 (transcription coregulator activity) and GO:0003682 (chromatin binding), overlapped with genes uniquely modified by H3K4me3 at TSS regions.

### Motif analysis

**FIGURE 5:**
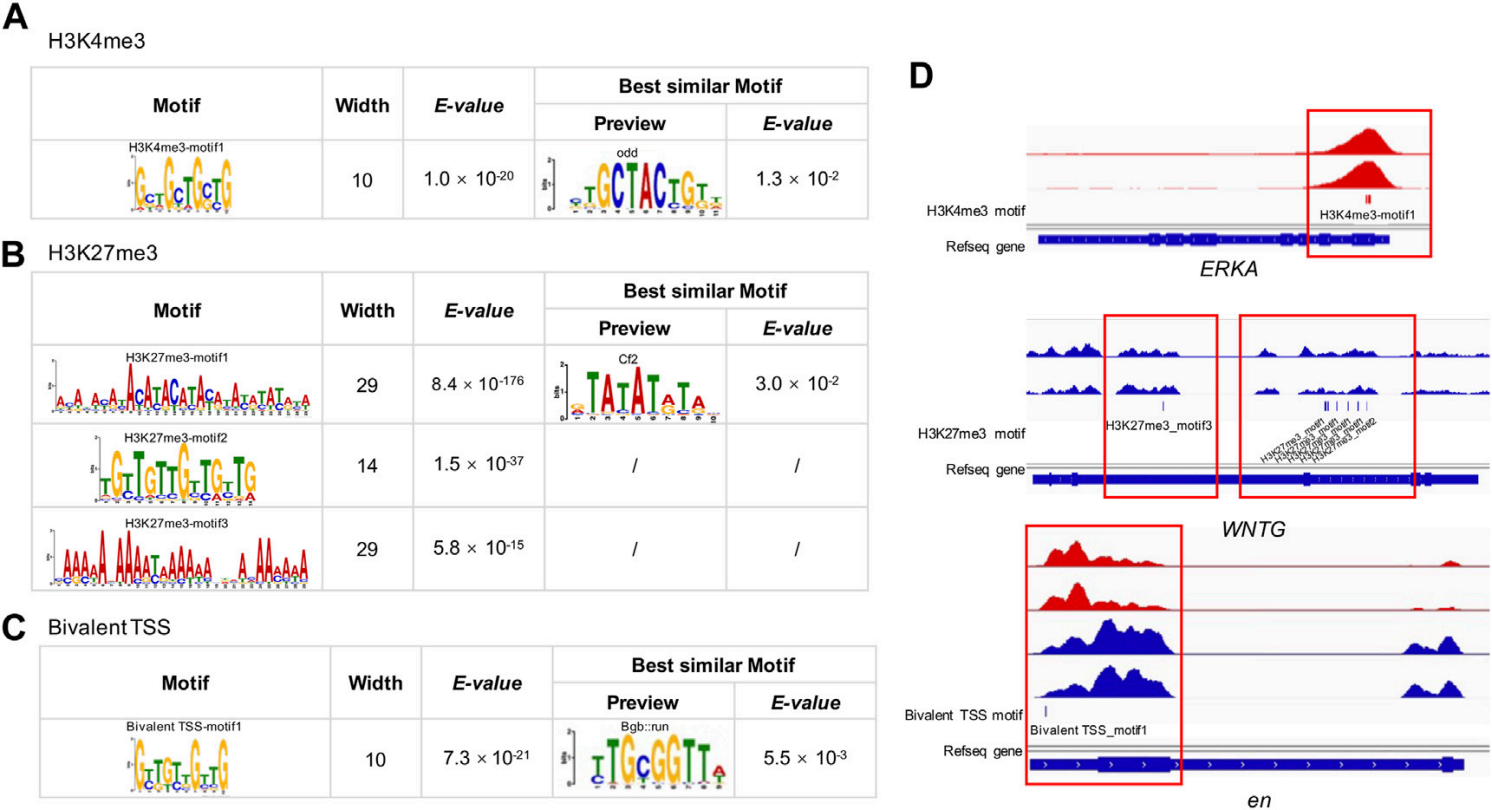
MEME-CHIP analysis of H3K4me3, H3K27me3 and bivalent domains motif in the *B. dorsalis* genome. Top consensus sequences identified by *de novo* motif discovery for **(A)** H3K4me3, **(B)** H3K27me3 and **(C)** bivalent TSSs, and the best similar known motifs previously described in *Drosophila*. **(D)** Examples of the locations of putative motifs within genes related to flight activity. See Supplementary Table S7 for motif position information for all genes related to flight activity.

We detected one, three and one significantly enriched motifs in the TSS regions of genes uniquely modified by H3K4me3, H3K27me3, and bivalent domains, respectively (Figures 5A–C; Supplementary Table S7). The significantly enriched motif for H3K4me3 was ‘GCTGCT’ (*E*-value = 1.0 × 10^−20^, frequency = 401), similar to the motif of odd (*E*-value = 1.3 × 10^−2^) identified in the JASPAR2018_CORE_insects database. The most enriched motif for H3K27me3 was ‘ACAT’ (*E*-value = 8.4 × 10^−176^, frequency = 73,968), which is similar to the motif of Cf2 (*E*-value = 3.0 × 10^−2^) in the database. The second most enriched motif for H3K27me3 was a TGT-rich motif (*E*-value = 1.5 × 10^−37^, frequency = 69,886). The third most significantly enriched motif was an AA-rich motif (*E*-value = 5.8 × 10^−15^, frequency = 4,139). The significantly enriched motif for bivalent domains was ‘GTTGTT’ (*E*-value = 7.3 × 10^−21^, frequency = 74), which is similar to the known motif of Bgb:run (*E*-value = 5.5 × 10^−3^).

### Potential regulatory role of histone modifications in flight activity

To explore the possible relevance of histone modification to flight activity, we analyzed the overlap between genes annotated with peaks and previously documented genes involved in wing development and migratory flight activity. We found 20 genes overlapped between peak-annotated genes and genes involved in the development of insect wings (*G* = 4.2, df = 1, *p* = 4 × 10^−2^, Supplementary Table S2), for example, *epidermal growth factor receptor* (*EGFR*), *wingless* (*wg*), *cubitus interruptyus* (*ci*), *decapentaplegic* (*dpp*), *hedgehog* (*hh*), including nine genes modified by H3K4me3, eight genes modified by H3K27me3, and three genes with bivalent promoters. Besides, we found 268 genes overlapped between peak-annotated genes and genes involved in migratory flight activity (Supplementary Table S2), including 55 metabolism related genes (*G* = 4.1, df = 1, *p* = 4 × 10^−2^), 14 genes related to environmental sensing, timing and navigation (*G* = 3.0, df = 1, *p* = 8 × 10^−2^), 15 genes involved in insulin signaling pathways (*G* = 12.3, df = 1, *p* = 5 × 10^−4^), 11 juvenile hormone (JH) and ecdysone related genes (*G* = 3.0, df = 1, *p* = 8 × 10^−2^), 5 genes associated with octopamine synthesis (*G* = 207.46, df = 1, *p* < 2.2 × 10^−16^), 10 genes associated with neuropeptide hormones (*G* = 3.3, df = 1, *p* = 8 × 10^−7^), 73 muscle function related genes (*G* = 20.4, df = 1, *p* = 6.3 × 10^−6^), 16 genes involved in transforming growth factor *β* signalling (*G* = 27.2, df = 1, *p* = 1.9 × 10^−7^), and 69 genes involved in JAK/STAT pathway and stress and immunity (*G* = 1.1, df = 1, *p* = 0.3). In total, the two hPTMs showed significant enrichment in 60% of the gene categories in Supplementary Table S2 when compared to expected by chance. As H3K4me3 and H3K27me3 are generally associated with actively transcribed genes and repressed genes, respectively (Benayoun et al., 2014; Aranda et al., 2015), we further found that 76.1% of H3K4me3-modified genes overlapped with upregulated genes, and 60.0% of H3K27me3-modified genes overlapped with downregulated genes involved in Supplementary Table S2. The widespread modification of genes relevant to wing development and migratory flight activity suggest a potentially key role of histone modifications in flight activity. When analyzing the relevance of motifs to wing development and metabolism, we found that 38.7% (29 out of 75) of the genes in the list of insect wings and muscle energy supply contained one or more putative DNA motifs (Figure 5D; Supplementary Table S7), suggesting the potential roles of these motifs in regulating insect flight activity.

## Discussion

The role of histone modification in regulating phenotypic responses to environmental change in model and non-invasive species has received increased attention in recent years (Simola et al., 2016; Kacsoh et al., 2017; Wojciechowski et al., 2018; Gong et al., 2021). However, the regulatory landscape and potential functional relevance of histone modification in invasive species remain poorly understood (Jeremias et al., 2018; Vogt, 2021). We used a quantitative, single-base-resolution technique (ChIP-seq) to profile genome-wide patterns of H3K4me3 and H3K27me3, which are two key histone modifications involved in gene regulation and phenotypic change, from thorax muscle tissue of a highly invasive insect, *B. dorsalis*. Despite a generally distinct genomic locations and functions between the two histone modifications, we identified a small number of overlapping regions possibly due to promoter bivalency. The combined analysis of ChIP-seq and RNA-seq data supports that H3K4me3 coincides with actively transcribed genes and H3K27me3 is associated with repressed genes that show low levels of transcription. Importantly, we found genes that are previously identified to be associated with wing development and migratory flight activity were largely modified by H3K4me3 and H3K27me3 in *B. dorsalis* (Supplementary Table S2), providing a support for the possible role of histone modification in flight activity. Finally, we identified one, three and one putative motifs for H3K4me3, H3K27me3 and bivalent TSSs modified regions, respectively that were also likely to be important for flight activity. Overall, our study provides the first genome-wide investigation of histone modifications in an invasive insect pest, and identifies genomic regions modified by histone markers that may be relevant to rapid range expansion of invasive insect species.

### Application of ChIP-seq on the non-model *B. dorsalis*

In this study, we present a protocol for preparing and analyzing H3K4me3 and H3K27me3 ChIP-seq data on a non-model invasive species, providing a powerful opportunity to examine the role of an under-explored epigenetic mechanism in insect that is likely to regulate invasiveness. The efficacy of our protocol was evaluated by a number of lines of evidence: 1) 40.2%–55.7% of reads were uniquely mapped to the reference genome, which is similar to the results from recent studies in other insects, for example, mosquito (whole body, 53.7%–58.2%) (Gómez-Díaz et al., 2014) and ant (brain, 17.2%–23.0%) (Simola et al., 2013). 2) Based on the uniquely mapped reads, we identified 7,226 H3K4me3 and 11,417 H3K27me3 peaks. While the number of H3K4me3 and H3K27me3 peaks identified in our study were smaller than the number of peaks found in *D. melanogaster*, mosquito (Gómez-Díaz et al., 2014; Ruiz et al., 2019) and silkworm (Cheng et al., 2018), and the genome size of *B. dorsalis* (468.7 Mb) is larger than *D. melanogaster* (138.9 Mb), mosquito (250.4 Mb) and silkworm (429.0 Mb), it could be due to the quality of the available genome annotation. 3) We found different distribution patterns between the two histone modifications, where H3K27me3 was more likely to be located within the intergenic regions, and H3K4me3 predominantly enriched in the genetic regions, in consistent with previous studies in yeast, plants, worms, flies and mammals (Bernstein et al., 2005; Liu et al., 2005; Zhang et al., 2009; Benayoun et al., 2014). 4) The combined analysis of ChIP-seq and RNA-seq data supports a relationship between transcription levels and profiles of histone modifications, with H3K4me3 coincides with actively transcribed genes, H3K27me3 is associated with repressed genes that show low levels of transcription, and the average expression level of genes not modified by hPTMs is lower than the average expression level of genes modified by H3K4me3 but higher than the genes modified by H3K27me3. 5) Gene annotation and ontology analysis of the two histone modifications showed the assumed expression of genes and an enrichment of GO terms expected to be found in muscle tissue. Taken together, the distribution patterns and functional annotations of H3K4me3 and H3K27me3 produced by our ChIP-seq protocol were comparable with the known results of histone modifications from other animal species, indicating strong reliability of our ChIP-seq data, and offering a proof-of-concept validation of our approach.

### Functional analyses of H3K4me3, H3K27me3, and bivalent modifications in *B. dorsalis*

Our functional analyses showed that H3K4me3 modifications were enriched around the TSSs and 5′end of genes, while H3K27me3 modifications were found in the entire body of genes, which are consistent with the distinct distribution patterns between these two histone modifications observed in *Drosophila* and mammals (Boyer et al., 2006; Lee et al., 2006; Eissenberg et al., 2007; Schuettengruber et al., 2009; Mohan et al., 2011), suggesting a conserved regulatory role of histone modifications across animal taxa. In addition, we found a clear difference between genes and functions annotated with the two histone modifications. The peaks of H3K4me3 overlapped with genes both encoding proteins relevant to general molecular regulation progress and physiologic processes, and specific functions in muscles such as energy metabolism process, suggesting an involvement of H3K4me3 in regulating both house-keeping genes essential to all tissues (Pan et al., 2007) and signaling pathways active in muscles (Brisson et al., 2007; Cao and Jiang, 2017). In contrast, genes annotated with H3K27me3 peaks were more likely to repress genes that are not expected to highly expressed in muscles, for example, *Oct-TyrR*, *Nlg2*, and *Nlg4* are genes associated with nervous system-related processes that were modified by H3K27me3, which was also found weakly expressed in adult *Drosophila* muscle tissue (Zhang et al., 2018). Taken together, these findings indicate that H3K4me3 likely regulates active transcription, while H3K27me3 is associated with transcriptional repression in *B. dorsalis*.

Despite a general mutually exclusive pattern of the genomic distributions and regulatory roles between H3K4me3 and H3K27me3, we also found bivalent TSS regions marked by both modifications. However, in contrast to previous finding that bivalent marks were widely distributed in the genomes of mammals and fish (Pan et al., 2007; Zhao et al., 2007; Vastenhouw et al., 2010; Sachs et al., 2013; Zhu et al., 2019; Blanco et al., 2020), and genes with bivalent promoters considered poised for expression (Mikkelsen et al., 2007; Voigt et al., 2013; Harikumar and Meshorer, 2015), we only identified 2.1% (139 out of 6,616) of all TSSs as bivalent TSSs in *B. dorsalis*, and the genes with bivalent TSSs were not in poised state (Gaertner et al., 2012). This is consistent with a recent findings in *Drosophila* that both marks were not significant coexistence in TSS regions (Schuettengruber et al., 2007; Schuettengruber et al., 2009; Gan et al., 2010; Schwartz and Cavalli, 2017), suggesting that bivalent domains might be restricted to certain organisms (Voigt et al., 2013). Such species-specific patterns have also been observed in DNA methylation, where vertebrate genomes are usually highly methylated while insect genomes are typically sparsely methylated (Roberts and Gavery, 2012). Because it is often suites of epigenetic mechanisms that act in concert to influence biological functions in animals, our finding suggests that the distribution and regulatory roles of bivalent modifications are phylogenetically unique.

### Motif analysis of H3K4me3 and H3K27me3 in *B. dorsalis*

To further characterize the functional role of the two histone modifications that cannot be explored in the regular peak calling analysis, we performed motif analysis, and identified a number of motifs for both marks and the bivalent domains within TSS regions, consistent with the binding preferences of known motifs important for *Drosophila* development (Fornes et al., 2020). For example, the ‘GCTGCT’ motifs, identified as the principal motifs of the H3K4me3 peak binding, was highly homologous to the odd motif in *Drosophila* (Nüsslein-Volhard and Wieschaus, 1980). odd encodes a zinc finger protein that represses other segmentation genes in the early *Drosophila* embryo (Ward and Coulter, 2000), and plays a key role in *wg* signalling (Cadigan et al., 1994; Beaven and Denholm, 2018). We also identified an important motif for H3K27me3 peaks, ‘ACAT’, which is homologous to Cf2 (Chorion factor 2) in *Drosophila*. Cf2 is a key regulator during embryo muscle formation, and participates in the regulation of the final size and number of nuclei present in skeletal, visceral and cardiac muscles (Bagni et al., 2002; Arredondo et al., 2017). Due to their highly functional relevance, this collection of motifs may be important for facilitating muscle development and structure maintenance, and warrant future studies to provide a more complete understanding of the functional link between muscle functions and the underlying motifs.

### Epigenetic regulation of flight activity in *B. dorsalis*

The strong flight ability along with its underlying physiological, morphological and behavioral mechanisms facilitating its expansion to new environments consist of a key component of invasiveness in *B. dorsalis* (Liang et al., 2001; Froerer et al., 2010). When comparing the overlap between a list of previously documented genes involved in wing development and migratory flight activity of *B. dorsalis* and peak-annotated genes, we found that a large number of these genes were modified by histone modifications in *B. dorsalis*, suggesting a possibly regulatory role of histone modification in flight activity. For example, we identified a number of overlapped genes with functions relevant to wing development, including *EGFR*, *wg*, *ci*, *dpp*, *hh*, *omb*, and *ubx* signaling pathways that have been found especially important in wing vein morphogenesis and structural basis for long-distance flight of insects (Strigini and Cohen, 2000; Roch et al., 2002; Zecca and Struhl, 2002; Brisson et al., 2010; Shen et al., 2010; Paul et al., 2013; Quah et al., 2015; Tomoyasu, 2017; Liu et al., 2020). Insect flight is the most energy demanding activity known in the animal species (Arrese and Soulages, 2010). In this study, we identified overlapped genes with functions relevant to muscle energy metabolism, for example, genes identified with roles in lipid metabolism, such as long-chain fatty-acid CoA ligase, desaturase related genes (Parisi et al., 2013; Jeffries et al., 2014), and carbohydrate metabolism genes, such as genes involved in trehalose transporter (Tret), glucosidase, maltase, hexokinase, glycogen phosphorylase (GlyP), and glycerol-3-phosphate dehydrogenase (Gpdh) (Barnes and Laurie-Ahlberg, 1986; Eanes et al., 2006; Kanamori et al., 2010; Inomata et al., 2019). Besides, we also identified overlapped genes related to PI3K signaling pathways that participate in redox reactions and the metabolism of lipids and carbohydrates (Nässel et al., 2013; Mattila and Hietakangas, 2017). Muscle assembly is focused in the thorax of flies and is primary associated with flight. We also identified a suite of overlapped genes associated with flight muscle structure, such as genes involved in lamins, Actin, sallimus (sls), flightin, and collagen (Hiromi and Hotta, 1985; Reedy et al., 2000; Bullard et al., 2005; Uchino et al., 2013). In addition, we annotated various genes involved in hormonal regulation, for example, insulin signaling pathway that regulates nutrient delivery and redistribution, which have been suggested playing a key role in wing formation and flight muscle development (Grönke and Partridge, 2010; Lin et al., 2016). Particularly, we found genes with bivalent TSSs that were relevant to JH signaling pathways, which has been suggested affecting flight, energy metabolism, and reproduction in insects (Flatt et al., 2005; Wang et al., 2012; Guo et al., 2018; Jones et al., 2018). In summary, our results suggest that histone modifications possibly play an important role in regulating genes relevant to flight activity in *B. dorsalis* (Roques et al., 2009; Guo et al., 2018; Jiang et al., 2022), which could provide the basis information for studies of epigenetic signatures of the flight activity of other invasive insects in the future study.

### Potential caveats

Our study has some limitations that should be noted. First, unlike model species such as human, mouse, worm and fly, there is no blacklist in non-model organisms that contains the regions of high signal that presumably represent unannotated repeats in the genome (Consortium, 2012; Boyle et al., 2014; Yue et al., 2014). Therefore, our results were inevitably influenced by the adverse effect of significant enrichment of signal from amplification of noise (Carroll et al., 2014). Second, tissue and developmental stage can influence the genome-wide histone modification landscape (Bonn et al., 2012; Jambhekar et al., 2019). We only used muscle tissue from sexually matured adult stage of *B. dorsalis*, preventing the generalization of our results to other tissues or developmental stage that may also be important to flight, especially migratory flight activity. Sampling during the initial stage of ovarian development, when the migratory flight behavior of migratory insect often occurs (Xiao et al., 2013), maybe give more information in the future study. Third, the flight activities of insects are underpinned by large modifications in gene regulation (Doyle et al., 2022). We only profiled two histone modifications that are typically found in promoters and coding regions, while a number of different histone marks, such as H3K27ac, H3K9me1 and H3K36me1 are known to be enriched in non-coding elements and are also functionally important (Zhou et al., 2011). In the future, considering multiple hPTMs and link them with specific flight capacity phenotype will further reveal the regulatory role of histone modifications in insect flight activities. In addition, it is important to compare the pattern of hPTMs in invasive vs. non-invasive species, which could provide more information on epigenetic regulation of invasiveness. The closely related *B. minax*, which has much more restricted host use and is less invasive compared to *B. dorsalis* could have been a relevant species for comparison in the future study. Thus, using ChIP-seq to profile additional histone modifications in more tissues and developmental stages will be helpful to provide a more comprehensive view of the regulatory landscape of histone modification and epigenetic architecture in *B. dorsalis*.

## Conclusion

Consistent with previous studies of model and endemic species, here we present the first genome-wide regulatory landscape of H3K4me3 and H3K27me3 histone modifications in the invasive *B. dorsalis*. We validated the efficacy of our ChIP-seq protocol in *B. dorsalis*, and found generally contrasting distributions of the two histone marks across the genome, with H3K4me3 mainly located downstream of the TSSs of genes, H3K27me3 tended to occupy broader regions covering the entire body of genes, and a small number of bivalent TSS regions modified by both histone modifications. Transcriptomic analysis supported a link between various histone modification profiles and transcription levels in *B. dorsalis*, with H3K4me3 associated with active gene transcription, and H3K27me3 is mostly associated with transcriptional repression. We also identified distinct GO enrichment patterns between the two histone modifications that are likely to reflect the distinct roles of H3K4me3 vs. H3K27me3 in regulating tissue development and structure maintenance. Importantly, we identified modified regions annotated with genes that overlapped with previously documented genes involving in wing development and migratory flight activity in *B. dorsalis*, suggesting a potential role of histone modifications in flight activity. Our work adds to the few studies using non-model, invasive insect species to test for the utilization of ChIP-seq to profile histone modification. Our results suggest that H3K4me3 and H3K27me3 could play an important role in facilitating phenotypic change during insect development as well as range expansion for invasive species.

## Data Availability

The original contributions presented in the study are publicly available. This data can be found here: https://www.ncbi.nlm.nih.gov/sra/PRJNA911513.
