# Supplementary material for: ChIP-seq profiling of H3K4me3 and H3K27me3 in an invasive insect, Bactrocera dorsalis
Source: Front Genet. 2023 Feb 23;14:1108104. doi: 10.3389/fgene.2023.1108104 (PMC9996634; doi:10.3389/fgene.2023.1108104)
Supplement: Supplementary file 1 [file DataSheet3.docx]

################## ChIP-seq analysis ##################

#######################################################

#### Part1. Trimming, filtering and mapping reads. ####

#######################################################

# Step1. Trimming the raw read files using Trim Galore! v0.6.6

trim_galore --illumina -o ./cleandata --paired ./Bd_Input_R1.fastq.gz ./Bd_Input_R2.fastq.gz

trim_galore --illumina -o ./cleandata --paired ./Bd_H3K4me3_1_R1.fastq.gz ./Bd_H3K4me3_1_R2.fastq.gz

trim_galore --illumina -o ./cleandata --paired ./Bd_H3K4me3_2_R1.fastq.gz ./Bd_H3K4me3_2_R2.fastq.gz

trim_galore --illumina -o ./cleandata --paired ./Bd_H3K27me3_1_R1.fastq.gz ./Bd_H3K27me3_1_R2.fastq.gz

trim_galore --illumina -o ./cleandata --paired ./Bd_H3K27me3_2_R1.fastq.gz ./Bd_H3K27me3_2_R2.fastq.gz

# Step2. Mapping the reads files using Bowtie2 v2.4.2

bowtie2 -x ./ref/genome.fasta -1 ./cleandata/Bd_Input_R1.fq.gz -2 ./cleandata/Bd_Input_R2.fq.gz 2>Input.mapping.metrics.txt | samtools view -o Input.bam

bowtie2 -x ./ref/genome.fasta -1 ./cleandata/Bd_H3K4me3_1_R1.fq.gz -2 ./cleandata/Bd_H3K4me3_1_R2.fq.gz 2>IP_1.mapping.metrics.txt | samtools view -o IP_1.bam

bowtie2 -x ./ref/genome.fasta -1 ./cleandata/Bd_H3K4me3_2_R1.fq.gz -2 ./cleandata/Bd_H3K4me3_2_R2.fq.gz 2>IP_2.mapping.metrics.txt | samtools view -o IP_2.bam

bowtie2 -x ./ref/genome.fasta -1 ./cleandata/Bd_H3K27me3_1_R1.fq.gz -2 ./cleandata/Bd_H3K27me3_1_R2.fq.gz 2>IP_3.mapping.metrics.txt | samtools view -o IP_3.bam

bowtie2 -x ./ref/genome.fasta -1 ./cleandata/Bd_H3K27me3_2_R1.fq.gz -2 ./cleandata/Bd_H3K27me3_2_R2.fq.gz 2>IP_4.mapping.metrics.txt | samtools view -o IP_4.bam

# Step3. Filtering the reads files using SAMtools v1.3.1, Picard v2.25.1, and deepTools v3.5.1

## 3.1 Filtering unmapped and non-uniquely mapped reads_Sort Aligned File

samtools sort -o Input.s.bam Input.bam

samtools sort -o IP_1.s.bam IP_1.bam

samtools sort -o IP_2.s.bam IP_2.bam

samtools sort -o IP_3.s.bam IP_3.bam

samtools sort -o IP_4.s.bam IP_4.bam

## 3.2 Filtering unmapped and non-uniquely mapped reads_Index Aligned File

samtools index Input.s.bam

samtools index IP_1.s.bam

samtools index IP_2.s.bam

samtools index IP_3.s.bam

samtools index IP_4.s.bam

## 3.3 Filtering unmapped and non-uniquely mapped reads_Filter

samtools view -F 3852 -o Input.af.bam Input.s.bam

samtools view -F 3852 -o IP_1.af.bam IP_1.s.bam

samtools view -F 3852 -o IP_2.af.bam IP_2.s.bam

samtools view -F 3852 -o IP_3.af.bam IP_3.s.bam

samtools view -F 3852 -o IP_4.af.bam IP_4.s.bam

# 3.4 Filtering improperly paired reads_Index Aligned File

samtools index Input.af.bam

samtools index IP_1.af.bam

samtools index IP_2.af.bam

samtools index IP_3.af.bam

samtools index IP_4.af.bam

# 3.5 Filtering improperly paired reads_Filter

samtools view -b -f 2 Input.af.bam -o Input.pf.bam

samtools view -b -f 2 IP_1.af.bam -o IP_1.pf.bam

samtools view -b -f 2 IP_2.af.bam -o IP_2.pf.bam

samtools view -b -f 2 IP_3.af.bam -o IP_3.pf.bam

samtools view -b -f 2 IP_4.af.bam -o IP_4.pf.bam

## 3.6 Filtering low quality reads_Index Aligned File

samtools index Input.pf.bam

samtools index IP_1.pf.bam

samtools index IP_2.pf.bam

samtools index IP_3.pf.bam

samtools index IP_4.pf.bam

## 3.7 Filtering low quality reads_Filter

samtools view -b -q 30 Input.pf.bam -o Input.qf.bam

samtools view -b -q 30 IP_1.pf.bam -o IP_1.qf.bam

samtools view -b -q 30 IP_2.pf.bam -o IP_2.qf.bam

samtools view -b -q 30 IP_3.pf.bam -o IP_3.qf.bam

samtools view -b -q 30 IP_4.pf.bam -o IP_4.qf.bam

## 3.8 Filtering PCR duplicates_Index Aligned File

samtools index Input.qf.bam

samtools index IP_1.qf.bam

samtools index IP_2.qf.bam

samtools index IP_3.qf.bam

samtools index IP_4.qf.bam

## 3.9 Filtering PCR duplicates_Filter

picard MarkDuplicates INPUT=Input.qf.bam OUTPUT=Input.df.bam METRICS_FILE=Input.df.metrics.txt VALIDATION_STRINGENCY=LENIENT REMOVE_DUPLICATES=true

picard MarkDuplicates INPUT=IP_1.qf.bam OUTPUT=IP_1.df.bam METRICS_FILE=IP_1.df.metrics.txt VALIDATION_STRINGENCY=LENIENT REMOVE_DUPLICATES=true

picard MarkDuplicates INPUT=IP_2.qf.bam OUTPUT=IP_2.df.bam METRICS_FILE=IP_2.df.metrics.txt VALIDATION_STRINGENCY=LENIENT REMOVE_DUPLICATES=true

picard MarkDuplicates INPUT=IP_3.qf.bam OUTPUT=IP_3.df.bam METRICS_FILE=IP_3.df.metrics.txt VALIDATION_STRINGENCY=LENIENT REMOVE_DUPLICATES=true

picard MarkDuplicates INPUT=IP_4.qf.bam OUTPUT=IP_4.df.bam METRICS_FILE=IP_4.df.metrics.txt VALIDATION_STRINGENCY=LENIENT REMOVE_DUPLICATES=true

## 3.10 Read counts were counts per million (CPM) normalized and converted to bigWig format_Index Aligned File

samtools index Input.df.bam

samtools index IP_1.df.bam

samtools index IP_2.df.bam

samtools index IP_3.df.bam

samtools index IP_4.df.bam

## 3.11 Read counts were CPM normalized and converted to bigWig format

bamCoverage -b Input.df.bam -o Input.df.bw -p 10 --normalizeUsing CPM --binSize 10 --extendReads 200

bamCoverage -b IP_1.df.bam -o IP_1.df.bw -p 10 --normalizeUsing CPM --binSize 10 --extendReads 200

bamCoverage -b IP_2.df.bam -o IP_2.df.bw -p 10 --normalizeUsing CPM --binSize 10 --extendReads 200

bamCoverage -b IP_3.df.bam -o IP_3.df.bw -p 10 --normalizeUsing CPM --binSize 10 --extendReads 200

bamCoverage -b IP_4.df.bam -o IP_4.df.bw -p 10 --normalizeUsing CPM --binSize 10 --extendReads 200

############################################################

#### Part2. Peak calling, annotation, and visualization ####

############################################################

# Step1. Peak calling using MACS2 v2.1.1

## 1.1 Peak calling for H3K4me3 and H3K27me3

macs2 callpeak -t ./IP_1.df.bam -c ./Input.df.bam -f BAM -n IP1 -g 4.53e8 --keep-dup all

macs2 callpeak -t ./IP_2.df.bam -c ./Input.df.bam -f BAM -n IP1 -g 4.53e8 --keep-dup all

macs2 callpeak -t ./IP_3.df.bam -c ./Input.df.bam -f BAM -n IP3 --broad -g 4.53e8 --broad-cutoff 0.1 --bdg --keep-dup all

macs2 callpeak -t ./IP_4.df.bam -c ./Input.df.bam -f BAM -n IP4 --broad -g 4.53e8 --broad-cutoff 0.1 --bdg --keep-dup all

## 1.2 Determining reproducible peaks between replicates using irreproducible discovery rate (IDR) for H3K4me3

macs2 callpeak -t ./IP_1.zf.bam ./IP_2.zf.bam -c ./Input.zf.bam -f BAM -n H3K4me3 -g 4.2e8 --keep-dup all -p 0.01

idr --samples IP1_peaks.narrowPeak IP2_peaks.narrowPeak --input-file-type narrowPeak --rank p.value --output-file H3K4me3_1-idr --plot --log-output-file H3K4me3_1.idr.log

cut -f 1-10 H3K4me3_1-idr | sort -k1,1 -k2,2n -k3,3n >H3K4me3.idr.narrowPeak

## 1.3 Determining reproducible peaks between replicates using the ‘findOverlapsOfPeaks’ function in the R package ChIPpeakAnno for H3K27me3

library(ChIPpeakAnno)

H3K27me3_1_peaks <- read.delim("H3K27me3_1_peaks.txt")

H3K27me3_2_peaks <- read.delim("H3K27me3_2_peaks.txt")

H3K27me3_1_peaks <- toGRanges(H3K27me3_1_peaks, format="broadPeak")

H3K27me3_2_peaks <- toGRanges(H3K27me3_2_peaks, format="broadPeak")

ol <- findOverlapsOfPeaks(H3K27me3_1_peaks, H3K27me3_2_peaks, connectedPeaks= "merge")

ol <- addMetadata(ol, colNames="score", FUN=mean)

makeVennDiagram(ol, fill=c("#009E73", "#F0E442"), # circle fill color

col=c("#D55E00", "#0072B2"), #circle border color

cat.col=c("#D55E00", "#0072B2"),connectedPeaks= "merge")

write.csv(ol$peaklist[["H3K27me3_1_peaks///H3K27me3_2_peaks"]],"overlaps_H3K27me3.broadPeak")

## 1.4 Determining bivalent domain

bedtools intersect -a ./H3K4me3.idr.narrowPeak -b ./overlaps_H3K27me3.broadPeak|awk '{if(($3-$2+1)>=100) print}'|sort -k1,1 -k2,2n>./bivalent_domain.txt

# Step2. Peak annotation using the R package ChIPseeker v1.26.2

library(ChIPseeker)

library(GenomicFeatures)

# build Txdb

gtfFile = "GCF_000789215.1_ASM78921v2_genomic.gtf"

db = makeTxDbFromGFF(gtfFile, format = "gtf")

saveDb(db, file="bd.txdb.sqlite")

# read ChIP-seq peak bed file

peak_H3K4me3 = readPeakFile("H3K4me3.idr.narrowPeak")

peak_H3K27me3 = readPeakFile("overlaps_H3K27me3.broadPeak")

# annotation

H3K4me3_peakAnno = annotatePeak(peak_H3K4me3,

tssRegion = c(-3000, 3000),

TxDb = db,

verbose = F)

H3K4me3_peakAnno.df = as.data.frame(H3K4me3_peakAnno)

H3K27me3_peakAnno = annotatePeak(peak_H3K27me3,

tssRegion = c(-3000, 3000),

TxDb = db,

verbose = F)

H3K27me3_peakAnno.df = as.data.frame(H3K27me3_peakAnno)

# Output results

write.table(H3K4me3_peakAnno, file = "H3K4me3_peakAnno.txt",sep = '\t', quote = FALSE, row.names = FALSE)

write.table(H3K27me3_peakAnno, file = "H3K27me3_peakAnno.txt",sep = '\t', quote = FALSE, row.names = FALSE)

#Plot the results

tiff("H3K4me3_peakAnno_3.tiff")

p1 = plotAnnoPie(H3K4me3_peakAnno)

dev.off()

tiff("H3K27me3_peakAnno_3.tiff")

p2 = plotAnnoPie(H3K27me3_peakAnno)

dev.off()

# Step3. Peak visualization using the R package ChIPseeker v1.26.2

library(ChIPseeker)

library(GenomicFeatures)

# build Txdb

gtfFile = "GCF_000789215.1_ASM78921v2_genomic.gtf"

db = makeTxDbFromGFF(gtfFile, format = "gtf")

saveDb(db, file="bd.txdb.sqlite")

# read ChIP-seq peak bed file

peak_H3K4me3 = readPeakFile("H3K4me3.idr.narrowPeak")

peak_H3K27me3 = readPeakFile("overlaps_H3K27me3.broadPeak")

head(peak_H3K4me3, 4)

head(peak_H3K27me3, 4)

# visualization for H3K4me3

promoter <- getPromoters(TxDb=db, upstream=3000, downstream=3000)

tagMatrix <- getTagMatrix(peak_H3K4me3, windows=promoter)

plotAvgProf(tagMatrix, xlim=c(-3000, 3000), conf = 0.95, resample = 1000)

# visualization for H3K27me3

plotPeakProf2(peak = peak_H3K27me3, upstream = rel(0.20), downstream = rel(0.20),

conf = 0.95, by = "gene", type = "body", nbin = 800,

TxDb = db, weightCol = "V5",ignore_strand = F)

######################################################################################

#### Part3. GO analysis for genes associated with Cluster 1-3 #####

#### and genes with H3K4me3-only, H3K27me3-only and bivalent domains at the TSSs,#####

#### and genes with H3K27me3 at the gene body regions. #####

######################################################################################

## R code for Gene Ontology term enrichment analysis using the R package clusterProfiler v4.0.5

library(clusterProfiler)

library(tidyverse)

library(cowplot)

library(dplyr)

library(ggnewscale)

library(ggplot2)

library(stringr)

## get gene IDs from peak-tss association table

genes_Cluster1 = read.csv("ass.Cluster1.csv")

genes_Cluster1 = as.character(genes_Cluster1$geneId)

genes_Cluster1 = unique(genes_Cluster1)

genes_Cluster2 = read.csv("ass.Cluster2.csv")

genes_Cluster2 = as.character(genes_Cluster2$geneId)

genes_Cluster2 = unique(genes_Cluster2)

genes_Cluster3 = read.csv("ass.Cluster3.csv")

genes_Cluster3 = as.character(genes_Cluster3$geneId)

genes_Cluster3 = unique(genes_Cluster3)

genes_H3K4me3_onlyTSS = read.csv("ass.H3K4me3_onlyTSS.csv")

genes_H3K4me3_onlyTSS = as.character(genes_H3K4me3_onlyTSS$geneId)

genes_H3K4me3_onlyTSS = unique(genes_H3K4me3_onlyTSS)

genes_H3K27me3_onlyTSS = read.csv("ass.H3K27me3_onlyTSS.csv")

genes_H3K27me3_onlyTSS = as.character(genes_H3K27me3_onlyTSS$geneId)

genes_H3K27me3_onlyTSS = unique(genes_H3K27me3_onlyTSS)

genes_H3K27me3_genebody = read.csv("ass.H3K27me3_genebody.csv")

genes_H3K27me3_genebody = as.character(genes_H3K27me3_genebody$geneId)

genes_H3K27me3_genebody = unique(genes_H3K27me3_genebody)

genes_bivalent = read.csv("ass.bivalent.csv")

genes_bivalent = as.character(genes_bivalent$geneId)

genes_bivalent = unique(genes_bivalent)

# read emapper

emapper = read.csv("GO_all.csv")

GO = select(emapper, GID, GO) %>%

separate_rows(GO, sep = ',') %>%

filter(str_detect(GO, 'GO'))

# read term2name

term2name = read.csv("go_term2name.csv")

# GO enrichment

term2gene_GO = data.frame(TERM=GO$GO, GENE=GO$GID)

## genes_Cluster1

enGO_genes_Cluster1 = enricher(genes_Cluster1,

TERM2GENE = term2gene_GO,

TERM2NAME = term2name,

qvalueCutoff = 0.05,

pAdjustMethod = "BH")

GO_genes_Cluster1_summary = as.data.frame(enGO_genes_Cluster1)

write.csv(GO_genes_Cluster1_summary,file = "GO_genes_Cluster1.csv")

barplot(enGO_genes_Cluster1, showCategory = 20, font.size = 20, x = "GeneRatio")

## genes_Cluster2

enGO_genes_Cluster2 = enricher(genes_Cluster2,

TERM2GENE = term2gene_GO,

TERM2NAME = term2name,

qvalueCutoff = 0.05,

pAdjustMethod = "BH")

GO_genes_Cluster2_summary = as.data.frame(enGO_genes_Cluster2)

write.csv(GO_genes_Cluster2_summary,file = "GO_genes_Cluster2.csv")

barplot(enGO_genes_Cluster2, showCategory = 20, font.size = 20, x = "GeneRatio")

## genes_Cluster3

enGO_genes_Cluster3 = enricher(genes_Cluster3,

TERM2GENE = term2gene_GO,

TERM2NAME = term2name,

qvalueCutoff = 0.05,

pAdjustMethod = "BH")

GO_genes_Cluster3_summary = as.data.frame(enGO_genes_Cluster3)

write.csv(GO_genes_Cluster3_summary,file = "GO_genes_Cluster3.csv")

barplot(enGO_genes_Cluster3, showCategory = 20, font.size = 20, x = "GeneRatio")

## genes_H3K4me3_onlyTSS

enGO_genes_H3K4me3_onlyTSS = enricher(genes_H3K4me3_onlyTSS,

TERM2GENE = term2gene_GO,

TERM2NAME = term2name,

qvalueCutoff = 0.05,

pAdjustMethod = "BH")

GO_genes_H3K4me3_onlyTSS_summary = as.data.frame(enGO_genes_H3K4me3_onlyTSS)

write.csv(GO_genes_H3K4me3_onlyTSS_summary,file = "GO_genes_H3K4me3_onlyTSS.csv")

barplot(enGO_genes_H3K4me3_onlyTSS, showCategory = 20, font.size = 20, x = "GeneRatio")

## genes_H3K27me3_onlyTSS

enGO_genes_H3K27me3_onlyTSS = enricher(genes_H3K27me3_onlyTSS,

TERM2GENE = term2gene_GO,

TERM2NAME = term2name,

qvalueCutoff = 0.05,

pAdjustMethod = "BH")

GO_genes_H3K27me3_onlyTSS_summary = as.data.frame(enGO_genes_H3K27me3_onlyTSS)

write.csv(GO_genes_H3K27me3_onlyTSS_summary,file = "GO_genes_H3K27me3_onlyTSS.csv")

barplot(enGO_genes_H3K27me3_onlyTSS, showCategory = 20, font.size = 20, x = "GeneRatio")

## genes_H3K27me3_genebody

enGO_genes_H3K27me3_genebody = enricher(genes_H3K27me3_genebody,

TERM2GENE = term2gene_GO,

TERM2NAME = term2name,

qvalueCutoff = 0.05,

pAdjustMethod = "BH")

GO_genes_H3K27me3_genebody_summary = as.data.frame(enGO_genes_H3K27me3_genebody)

write.csv(GO_genes_H3K27me3_genebody_summary,file = "GO_genes_H3K27me3_genebody.csv")

barplot(enGO_genes_H3K27me3_genebody, showCategory = 20, font.size = 20, x = "GeneRatio")

## genes_bivalent

enGO_genes_bivalent = enricher(genes_bivalent,

TERM2GENE = term2gene_GO,

TERM2NAME = term2name,

qvalueCutoff = 0.05,

pAdjustMethod = "BH")

GO_genes_bivalent_summary = as.data.frame(enGO_genes_bivalent)

write.csv(GO_genes_bivalent_summary,file = "GO_genes_bivalent.csv")

barplot(enGO_genes_bivalent, showCategory = 20, font.size = 20, x = "GeneRatio")

################################

#### Part4. Motif analysis #####

################################

## H3K4me3

sort -k8,8nr ./H3K4me3.idr.narrowPeak | head -n 500 | awk -v OFS="\t" '{print $1,$2+$10,$2+$10+1}' >summit.bed

awk -v OFS="\t" '{print $1,$2-50,$2+50}' summit.bed | awk '$2>=0' >summit.l50.r50.bed

bedtools getfasta -fi ./ref/GCF_000789215.1_ASM78921v2_genomic.fna -bed summit.l50.r50.bed >summit.l50.r50.fa

## download motif database

wget http://meme-suite.org/meme-software/Databases/motifs/motif_databases.12.19.tgz

tar -xzf motif_databases.12.19.tgz

meme-chip -o summit.l50.r50.meme-chip -db ./motif_databases/JASPAR/JASPAR2018_CORE_insects_non-redundant.meme -seed 10 -meme-minw 8 -meme-maxw 10 -ccut 0 summit.l50.r50.fa

tomtom -oc summit.l50.r50.meme-chip.motif0.tomtom -min-overlap 5 -bfile ./summit.l50.r50.meme-chip/background ./summit.l50.r50.meme-chip/combined.meme motif_databases/FLY/fly_factor_survey.meme

bedtools getfasta -fi ./ref/GCF_000789215.1_ASM78921v2_genomic.fna -bed ./H3K4me3.idr.narrowPeak >peak.fa

fimo --parse-genomic-coord --oc summit.l50.r50.meme-chip.motif0.fimo --bgfile summit.l50.r50.meme-chip/background ./summit.l50.r50.meme-chip/combined.meme peak.fa

## H3K27me3

sort -k8,8nr ./overlaps_H3K27me3.broadPeak | head -n 500 | awk -v OFS="\t" '{print $1,$2+$10,$2+$10+1}' >summit.bed

awk -v OFS="\t" '{print $1,$2-50,$2+50}' summit.bed | awk '$2>=0' >summit.l50.r50.bed

bedtools getfasta -fi ./ref/GCF_000789215.1_ASM78921v2_genomic.fna -bed summit.l50.r50.bed >summit.l50.r50.fa

## download motif database

wget http://meme-suite.org/meme-software/Databases/motifs/motif_databases.12.19.tgz

tar -xzf motif_databases.12.19.tgz

meme-chip -o summit.l50.r50.meme-chip -db ./motif_databases/JASPAR/JASPAR2018_CORE_insects_non-redundant.meme -seed 10 -ccut 0 summit.l50.r50.fa

tomtom -oc summit.l50.r50.meme-chip.motif0.tomtom -min-overlap 5 -bfile ./summit.l50.r50.meme-chip/background ./summit.l50.r50.meme-chip/combined.meme motif_databases/FLY/fly_factor_survey.meme

bedtools getfasta -fi ./ref/GCF_000789215.1_ASM78921v2_genomic.fna -bed ./overlaps_H3K27me3.broadPeak >peak.fa

fimo --parse-genomic-coord --oc summit.l50.r50.meme-chip.motif0.fimo --bgfile summit.l50.r50.meme-chip/background ./summit.l50.r50.meme-chip/combined.meme peak.fa

## bivalent domains

sort -k8,8nr ./bivalent_domain.txt | head -n 500 | awk -v OFS="\t" '{print $1,$2+$10,$2+$10+1}' >summit.bed

awk -v OFS="\t" '{print $1,$2-50,$2+50}' summit.bed | awk '$2>=0' >summit.l50.r50.bed

bedtools getfasta -fi ./ref/GCF_000789215.1_ASM78921v2_genomic.fna -bed summit.l50.r50.bed >summit.l50.r50.fa

## download motif database

wget http://meme-suite.org/meme-software/Databases/motifs/motif_databases.12.19.tgz

tar -xzf motif_databases.12.19.tgz

meme-chip -o summit.l50.r50.meme-chip -db ./motif_databases/JASPAR/JASPAR2018_CORE_insects_non-redundant.meme -seed 10 -meme-minw 8 -meme-maxw 10 -ccut 0 summit.l50.r50.fa

tomtom -oc summit.l50.r50.meme-chip.motif0.tomtom -min-overlap 5 -bfile ./summit.l50.r50.meme-chip/background ./summit.l50.r50.meme-chip/combined.meme motif_databases/FLY/fly_factor_survey.meme

bedtools getfasta -fi ./ref/GCF_000789215.1_ASM78921v2_genomic.fna -bed ./overlaps_H3K27me3.broadPeak >peak.fa

fimo --parse-genomic-coord --oc summit.l50.r50.meme-chip.motif0.fimo --bgfile summit.l50.r50.meme-chip/background ./summit.l50.r50.meme-chip/combined.meme peak.fa

################## RNA-seq analysis ##################

#######################################################

#### Part1. Trimming, filtering and mapping reads. ####

#######################################################

# Step1. Trimming the raw read files using Trim Galore! v0.6.6

trim_galore --illumina -o ./cleandata --paired ./Bd_1_R1.fastq.gz ./Bd_1_R2.fastq.gz

trim_galore --illumina -o ./cleandata --paired ./Bd_2_R1.fastq.gz ./Bd_2_R2.fastq.gz

trim_galore --illumina -o ./cleandata --paired ./Bd_3_R1.fastq.gz ./Bd_3_R2.fastq.gz

# Step2. Mapping the reads files using HISAT2 v2.1.0

## 2.1 Building a HISAT2 index

hisat2-build -f ./ref/genome.fasta ./ref/genome 1>hisat2-build.log 2>&1

## 2.2 Mapping the reads files

hisat2 --new-summary -p 10 -k 1 --rna-strandness RF -x ./ref/genome -1 ./cleandata/Bd_1_R1_val_1.fq.gz -2 ./cleandata/Bd_1_R2_val_2.fq.gz -S Bd_1.sam 1>Bd_1.log 2>&1

hisat2 --new-summary -p 10 -k 1 --rna-strandness RF -x ./ref/genome -1 ./cleandata/Bd_2_R1_val_1.fq.gz -2 ./cleandata/Bd_2_R2_val_2.fq.gz -S Bd_2.sam 1>Bd_2.log 2>&1

hisat2 --new-summary -p 10 -k 1 --rna-strandness RF -x ./ref/genome -1 ./cleandata/Bd_3_R1_val_1.fq.gz -2 ./cleandata/Bd_3_R2_val_2.fq.gz -S Bd_3.sam 1>Bd_3.log 2>&1

# Step3. Sorting reads and building index using SAMtools v1.3.1

## 3.1 Sorting reads

samtools sort -o Bd_1.bam Bd_1.sam

samtools sort -o Bd_2.bam Bd_2.sam

samtools sort -o Bd_3.bam Bd_3.sam

## 3.2 Building index

samtools index Bd_1.bam

samtools index Bd_2.bam

samtools index Bd_3.bam

# Step4. Filtering the reads files using Picard v2.25.1

## 4.1

samtools sort -n -o Bd_1_namesort.bam Bd_1.bam

samtools sort -n -o Bd_2_namesort.bam Bd_2.bam

samtools sort -n -o Bd_3_namesort.bam Bd_3.bam

## 4.2

samtools fixmate Bd_1_namesort.bam Bd_1_fixmate.bam

samtools fixmate Bd_2_namesort.bam Bd_2_fixmate.bam

samtools fixmate Bd_3_namesort.bam Bd_3_fixmate.bam

## 4.3

samtools sort -o Bd_1_positionsort.bam Bd_1_fixmate.bam

samtools sort -o Bd_2_positionsort.bam Bd_2_fixmate.bam

samtools sort -o Bd_3_positionsort.bam Bd_3_fixmate.bam

## 4.4

samtools rmdup -S Bd_1_positionsort.bam Bd_1_markdup.bam

samtools rmdup -S Bd_2_positionsort.bam Bd_2_markdup.bam

samtools rmdup -S Bd_3_positionsort.bam Bd_3_markdup.bam

#######################################################

#### Part2. Quantifying the reads count. ####

#######################################################

htseq-count -f bam -r name -s reverse -a 10 -t exon -i gene_id -m union ./Bd_1_markdup.bam ./ref/genome.gtf > Bd_1_htseq.txt

htseq-count -f bam -r name -s reverse -a 10 -t exon -i gene_id -m union ./Bd_2_markdup.bam ./ref/genome.gtf > Bd_2_htseq.txt

htseq-count -f bam -r name -s reverse -a 10 -t exon -i gene_id -m union ./Bd_3_markdup.bam ./ref/genome.gtf > Bd_3_htseq.txt
