# Supplementary material for: ChIP-seq profiling of H3K4me3 and H3K27me3 in an invasive insect, Bactrocera dorsalis
Source: Front Genet. 2023 Feb 23;14:1108104. doi: 10.3389/fgene.2023.1108104 (PMC9996634; doi:10.3389/fgene.2023.1108104)
Supplement: Supplementary file 2 [file DataSheet1.docx]

**Supplemental Information 1 for:**

**ChIP-seq profiling of H3K4me3 and H3K27me3 in an invasive insect, *Bactrcocera dorsalis***

Yan Zhao1†, Juntao Hu2†, Jiajiao Wu3, Zhihong Li1*

1 College of Plant Protection, China Agricultural University, Beijing, 100193, PR China

2 Ministry of Education Key Laboratory for Biodiversity Science and Ecological Engineering, Institute of Biodiversity Science, Centre of Evolutionary Biology, School of Life Sciences, Fudan University, Shanghai, 200438, PR China

3 Technology Centre of Guangzhou Customs, Guangzhou, 510623, PR China

† These authors contributed equally to this work.

*Correspondence author: Zhihong Li ([lizh@cau.edu.cn](mailto:lizh@cau.edu.cn)).

**Table of Contents:**

| **Figure S1** | Page 2 |
| --- | --- |
| **Figure S2** | Page 3 |
| **Figure S3** | Page 4 |
| **Figure S4** | Page 5 |
| **Figure S5** | Page 6 |
| **Figure S6** | Page 7 |
| **Table S1** | Page 8 |
| **Table S2** | Page 9-18 |
| **Table S3** | Page 19 |
| **References** | Page 20-26 |

**Figure S1**. Quality evaluation of chromatin immunoprecipitation experiment. (A) Agarose gel electrophoresis of ChIP DNA before (Lane1) and after sonication (Lane 2) in the chromatin immunoprecipitation experiment. (B) Western blotting results of the availability of antibodies for H3K4me3 (ab8580, Abcam) and H3K27me3 (ab6002, Abcam) used in our study. (C) The final concentration and amount of DNA used for ChIP-seq library preparation.

**Figure S2**. The proportion of the reads after each stage of filtering. (A, B) The filtering results for the two H3K4me3 replicates. (C, D) The filtering results for the two H3K27me3 replicates. (E) The filtering results for the input.

**Figure S3**. Assessment of reads reproducibility. (A) Pearson correlation coefficient (PCC) and Spearman’s correlation coefficient (SCC) of the mapped read counts between H3K4me3 and H3K27me3 replicates. (B) Browser tracks of the input and ChIP-seq replicates.

**Figure S4**. Quality control of ChIP-seq data. (A) Quality metrics for assessing library complexity of the input, and H3K4me3 and H3K27me3 replicates. (B) Cross correlation analysis for H3K4me3 and H3K27me3. (C-D) ChIP enrichment (FRiP) and called regions of H3K4me3 and H3K27me3.

**Figure S5**. (A-D) Irreproducible discovery rate (IDR) plot showing the correspondence between replicates and the threshold for filtering for the H3K4me3 ChIP-Seq. (A) Replicate 1 peak ranks versus Replicate 2 peak ranks. Peaks not passing the specified IDR threshold are in red. (B) Log10 peak scores in Replicate 1 versus Replicate 2. Peaks not passing the specified IDR threshold are in red. (C-D) Peak rank versus IDR scores are plotted in black. Boxplots show the distribution of IDR values in each 5% quantile. (E) Venn diagram of overlaps for the number of peaks of two replicates for the H3K37me3 using the ‘findOverlapsOfPeaks’ function in the R package ChIPpeakAnno.

**Figure S6**. Cluster analysis of H3K4me3_2 and H3K27me3_2 in the *B. dorsalis* genome. (A) Heatmap and (B) density profile of the *K*-means clustering of mapped read position for H3K4me3_2 and H3K27me3_2 around TSS regions (± 3 kb) in the *B. dorsalis* genome.

**Table S1.** Ingredient of artificial diet for *Bactrocera dorsalis* larvae.

| **Name** | **Manufacture** | **Reagent dosage** |
| --- | --- | --- |
| sucrose | XIHUA, China | 125 g |
| wheat bran | Tianlong, China | 235 g |
| yeast extract | ANGEL YEAST, China | 31 g |
| sorbic acid | AOBOX, China | 1 g |
| methyl-p-hydroxybenzoate | HUSHI, China | 0.6 g |
| L-Ascorbic acid | HUSHI, China | 0.5 g |
| ddH_2_O | / | 600 mL |

**Table S2**. List of genes relevant to insect flight activity modified by H3K4me3-only, H3K27me3-only and bivalent modifications at TSSs, or by H3K27me3 at gene body regions in *Bactrocera dorsalis*.

| Process involved | Gene ID | Fold-Change | Gene name | Signaling pathway/Gene category involved | Citation |
| --- | --- | --- | --- | --- | --- |
| 1.Wing development | H3K4me3-only TSS | | | | |
|  | LOC105226382 | 30.04 | lateral signaling target protein 2 homolog/*LST2* | *epidermal growth factor receptor* (*EGFR*) | Roch et al., 2002; Zecca & Struhl, 2002 |
|  | LOC105223962 | 16.87 | protein wntless/*Wls* | *wingless* (*wg*) | Strigini & Cohen, 2000 |
|  | LOC105229790 | 36.19 | armadillo segment polarity protein/*ARM* |  |  |
|  | LOC105227911 | 11.24 | transcriptional activator cubitus interruptus/*CI* | *cubitus interruptyus* (*ci*) | Quah et al., 2015 |
|  | LOC105232021 | 15.77 | mothers against decapentaplegic homolog 3/*SMAD3* | *decapentaplegic* (*dpp*) | Paul et al., 2013 |
|  | LOC105229221 | 17.8 | mothers against decapentaplegic homolog 6/*SMAD6* |  |  |
|  | LOC105233589 | 18.05 | NPC intracellular cholesterol transporter 1/*Npc1* | *hedgehog* (*hh*) | Brisson et al., 2010 |
|  | LOC105226668 | 7.05 | protein patched/*PTC* |  |  |
|  | LOC105231715 | 17.14 | protein dispatched/*Disp* |  |  |
|  | H3K27me3-only TSS | | | | |
|  | LOC105226487 | 3.53 | protein giant-lens | *EGFR* | Roch et al., 2002; Zecca & Struhl, 2002 |
|  | LOC105225549 | 3.54 | protein wingless/*WNTG* | *wg* | Strigini & Cohen, 2000 |
|  | LOC105227616 | 3.62 | protein hedgehog/*HH* | *hh* | Brisson et al., 2010 |
|  | LOC105223015 | 2.77 | NPC intracellular cholesterol transporter 1 homolog 1b/Npc1 |  |  |
|  | LOC105225122 | 3.04 | protein decapentaplegic/Dpp | *dpp* | Paul et al., 2013 |
|  | LOC105232239 | 6.61 | optomotor-blind protein | *optomotor blind* (*omb*) | Shen et al., 2010 |
|  | LOC105232240 | 6.31 | optomotor-blind protein |  |  |
|  | LOC105226092 | 3.79 | homeotic protein ultrabithorax/UBX | *ultrabithorax* (*ubx*) | Liu et al., 2020; Tomoyasu, 2017 |
|  | Bivalent TSS Fold-Change (H3K4me3/H3K27me3) | | | | |
|  | LOC105229692 | 5.30/4.51 | protein vestigial/*VG* | *vestigial* (*vg*) | Clark-Hachtel et al., 2013; Niwa et al., 2010 |
|  | LOC105233061 | 5.98/3.58 | protein apterous | *apterous* (*ap*) | Tomoyasu et al., 2017 |
|  | LOC105233941 | 3.40/7.30 | segmentation polarity homeobox protein engrailed/HMEN | *engrailed* (*en*) | O’Donnell & Jockusch, 2010 |
| 2. Metabolism | H3K4me3-only TSS | | | | |
|  | LOC105232303 | 4.87797 | very long-chain-fatty-acid--CoA ligase bubblegum | long-chain fatty-acid CoA ligase | Jeffries et al., 2014 |
|  | LOC105231732 | 33.7123 | acyl-CoA Delta(11) desaturase | Desaturase | Parisi et al., 2013 |
|  | LOC105232584 | 7.09102 | acyl-CoA Delta(11) desaturase |  |  |
|  | LOC105227657 | 80.5399 | facilitated trehalose transporter Tret1-2 homolog | *Trehalose transporter* (*Tret*) | Kanamori et al., 2010 |
|  | LOC105228649 | 39.964 | facilitated trehalose transporter Tret1-2 homolog |  |  |
|  | LOC105228750 | 3.2932 | trehalose-phosphate phosphatase B |  |  |
|  | LOC105228751 | 24.3491 | probable trehalose-phosphate phosphatase C |  |  |
|  | LOC105229407 | 43.2033 | facilitated trehalose transporter Tret1 |  |  |
|  | LOC105224497 | 205.33 | Glucosidase II beta subunit-like | *Glucosidase* | Inomata et al., 2019 |
|  | LOC105226933 | 202.719 | mannosyl-oligosaccharide glucosidase |  |  |
|  | LOC105231207 | 149.642 | endoplasmic reticulum lectin 1 |  |  |
|  | LOC105225131 | 221.411 | glucosidase 2 subunit beta |  |  |
|  | LOC105228045 | 31.4215 | maltase A3 | *Maltase* |  |
|  | LOC105229200 | 10.9832 | maltase 2 |  |  |
|  | LOC105221945 | 6.4496 | hexokinase type 2 | *Hexokinase* | Eanes et al., 2006 |
|  | LOC105222009 | 123.538 | glycogen phosphorylase | *Glycogen phosphorylase* (*GlyP*) | Eanes et al., 2006 |
|  | LOC105222824 | 82.3844 | glycerol-3-phosphate acyltransferase 1, mitochondrial | *Glycerol-3-phosphate dehydrogenase* (*Gpdh*) | Barnes & Laurie-Ahlberg, 1986 |
|  | LOC105226672 | 11.8274 | 1-acyl-sn-glycerol-3-phosphate acyltransferase alpha |  |  |
|  | LOC105223261 | 55.0359 | 1-acyl-sn-glycerol-3-phosphate acyltransferase gamma |  |  |
|  | LOC105223263 | 13.6831 | 1-acyl-sn-glycerol-3-phosphate acyltransferase gamma |  |  |
|  | LOC105224026 | 7.01822 | glycerol-3-phosphate phosphatase |  |  |
|  | LOC105224028 | 56.8256 | glycerol-3-phosphate phosphatase |  |  |
|  | LOC105225662 | 60.3283 | glycerol-3-phosphate dehydrogenase, mitochondrial |  |  |
|  | LOC105226401 | 82.4812 | glycerol-3-phosphate dehydrogenase [NAD(+)], cytoplasmic |  |  |
|  | LOC105228129 | 110.644 | glycerol-3-phosphate acyltransferase 4 |  |  |
|  | LOC105231866 | 180.242 | CDP-diacylglycerol--glycerol-3-phosphate 3-phosphatidyltransferase, mitochondrial |  |  |
|  | LOC105224744 | 9.65 | phosphatidylinositol 3-kinase 2/*FXL16* | Phosphatidylinositol-3-Kinase (PI3K) | Mattila & Hietakangas, 2017; Nässel et al., 2013 |
|  | LOC105224763 | 20.75 | phosphatidylinositol 4-phosphate 3-kinase C2 domain-containing subunit beta/*PIK3C2B* |  |  |
|  | LOC105225446 | 41.81 | phosphatidylinositol 4-phosphate 5-kinase type-1 alpha/*PI51A* |  |  |
|  | LOC105227287 | 27.11 | phosphatidylinositol 3-kinase catalytic subunit type 3/*PK3C3* |  |  |
|  | LOC105228519 | 13.77 | phosphatidylinositol 4-kinase type 2-beta/*P4K2B* |  |  |
|  | LOC105229287 | 14.55 | phosphatidylinositol 3-kinase regulatory subunit gamma/*P55G* |  |  |
|  | LOC105224919 | 20.6 | phosphatidylinositol 5-phosphate 4-kinase type-2 alpha/*PI42B* |  |  |
|  | LOC105233251 | 12.36 | phosphatidylinositol 4,5-bisphosphate 3-kinase catalytic subunit/*PK3CD* |  |  |
|  | LOC105222090 | 13.54 | mitogen-activated protein kinase kinase kinase 4/M3K4 | mitogen-activated protein kinases (MAPKs) | Li et al., 2012; Trempolec et al., 2013 |
|  | LOC105233339 | 13.37 | mitogen-activated protein kinase p38b |  |  |
|  | LOC105233323 | 33.69 | mitogen-activated protein kinase kinase kinase 7/M3K7 |  |  |
|  | LOC105224141 | 17.45 | dual specificity mitogen-activated protein kinase kinase dSOR1/DSOR1 |  |  |
|  | LOC105224414 | 6.52 | mitogen-activated protein kinase-binding protein 1/MABP1 |  |  |
|  | LOC105222491 | 20.4 | dual specificity mitogen-activated protein kinase kinase 4/MP2K4 |  |  |
|  | LOC105223761 | 24.19 | mitogen-activated protein kinase ERK-A/ERKA |  |  |
|  | LOC105224286 | 2.63 | mitogen-activated protein kinase kinase kinase 13-B |  |  |
|  | LOC105226954 | 22.31 | dual specificity mitogen-activated protein kinase kinase 6/MP2K6 |  |  |
|  | LOC105227595 | 13.29 | mitogen-activated protein kinase p38a/MK14A |  |  |
|  | LOC105228109 | 15.19 | mitogen-activated protein kinase kinase kinase kinase 5/M4K5 |  |  |
|  | LOC105230906 | 17.97 | mitogen-activated protein kinase kinase kinase 15/M3K5 |  |  |
|  | LOC105232152 | 15.34 | dual specificity mitogen-activated protein kinase kinase hemipterous/HEP |  |  |
|  | H3K27me3-only TSS | | | | |
|  | LOC105232306 | 2.601985 | long-chain-fatty-acid--CoA ligase bubblegum-like | long-chain fatty-acid CoA ligase | Jeffries et al., 2014 |
|  | LOC105229207 | 2.87803 | protein-glucosylgalactosylhydroxylysine glucosidase | *Glucosidase* | Inomata et al., 2019 |
|  | LOC105228202 | 2.638625 | maltase A2-like | *Maltase* |  |
|  | H3K27me3-body | | | | |
|  | LOC105232304 | 2.574615 | long-chain-fatty-acid--CoA ligase bubblegum-like | long-chain fatty-acid CoA ligase | Jeffries et al., 2014 |
|  | LOC105232306 | 2.713415 | long-chain-fatty-acid--CoA ligase bubblegum-like |  |  |
|  | LOC105231305 | 2.943753333 | facilitated trehalose transporter Tret1 | Trehalose transporter (Tret) | Kanamori et al., 2010 |
|  | LOC105222836 | 6.911335 | facilitated trehalose transporter Tret1-2 homolog |  |  |
|  | LOC105228109 | 2.68 | mitogen-activated protein kinase kinase kinase kinase 5/M4K5 | MAPKs | Li et al., 2012; Trempolec et al., 2013 |
| 3.Environmental sensing, timing and navigation | H3K4me3-only TSS | | | | |
|  | LOC105232494 | 7.05845 | general odorant-binding protein 99a | *Odorant-binding protein* (*Obp*) | Wang et al., 2020 |
|  | LOC105232791 | 5.14474 | general odorant-binding protein 70 |  |  |
|  | LOC105223236 | 11.2712 | neprilysin-4 | *neprilysin* (*NEP*) family | Rose et al., 2009; Turrel et al., 2016 |
|  | LOC105229617 | 7.55824 | neprilysin-2 |  |  |
|  | H3K27me3-only TSS | | | | |
|  | LOC105221869 | 2.98897 | general odorant-binding protein 56h | *Obp* | Wang et al., 2020 |
|  | LOC105222691 | 2.73951 | general odorant-binding protein 84a |  |  |
|  | LOC105228510 | 2.241245 | general odorant-binding protein 84a |  |  |
|  | LOC105229973 | 2.643765 | probable serine/threonine-protein kinase clkA |  |  |
|  | LOC105230135 | 2.83839 | general odorant-binding protein 56h |  |  |
|  | LOC105232339 | 2.60448 | neprilysin-4 | *NEP* family | Rose et al., 2009; Turrel et al., 2016 |
|  | LOC105232824 | 2.532665 | neprilysin-11 |  |  |
|  | H3K27me3-body | | | | |
|  | LOC105223031 | 3.22672 | neprilysin-1 | *NEP* family | Rose et al., 2009; Turrel et al., 2016 |
|  | LOC105232339 | 2.32716 | neprilysin-4 |  |  |
|  | LOC105232824 | 3.170295 | neprilysin-11 |  |  |
| 4.Insulin/insulin-like growth factor signalling | H3K4me3-only TSS | | | | |
|  | LOC105225647 | 38.61 | *Frs2* (insulin receptor binding) | insulin | (Grönke & Partridge 2010; Lin et al. 2016) |
|  | LOC105230971 | 7.26 | insulin-like receptor/*INSR* |  |  |
|  | LOC105233742 | 13.89 | forkhead box protein O/FoxO |  |  |
|  | LOC105227909 | 23.2 | insulin-degrading enzyme/*Ide* |  |  |
|  | LOC105228180 | 20.6 | *DOK2* (insulin receptor binding) |  |  |
|  | H3K27me3-only TSS | | | | |
|  | LOC105232307 | 2.46 | insulin-like growth factor-binding protein complex acid labile subunit/*ALS* | insulin | Grönke & Partridge 2010; Lin et al. 2016 |
|  | LOC105223506 | 4.71 | insulin gene enhancer protein isl-1/*ISL1* |  |  |
|  | LOC105230018 | 2.66 | *ALS* |  |  |
|  | LOC105232101 | 3.78 | insulin-like growth factor 2 mRNA-binding protein 1/*IF2B1* |  |  |
|  | H3K27me3-body | | | | |
|  | LOC105224097 | 2.63 | insulin-like growth factor-binding protein complex acid labile subunit/LRRN2 | insulin | Grönke & Partridge 2010; Lin et al. 2016 |
|  | LOC105232101 | 5.71 | *IF2B1* |  |  |
|  | LOC105230971 | 3.61 | insulin-like receptor/*INSR* |  |  |
|  | LOC105232307 | 2.71 | *ALS* |  |  |
|  | LOC105230018 | 2.66 | *ALS* |  |  |
|  | LOC105223506 | 4.71 | insulin gene enhancer protein isl-1/ISL1 |  |  |
| 5.Hormonal control: juvenile hormone and ecdysone related genes | H3K4me3-only TSS | | | | |
|  | LOC105230714 | 3.512 | juvenile hormone epoxide hydrolase 2-like | *juvenile hormone* (*JH*) | (Flatt et al., 2005; Jones et al., 2018; Wang et al., 2012 |
|  | LOC105222533 | 254.8 | SAGA-associated factor 11 homolog | ecdysone | Lin et al., 2018 |
|  | LOC105229606 | 126.178 | ras-like protein 1 |  |  |
|  | LOC105230060 | 125.459 | protein ecdysoneless |  |  |
|  | LOC105230085 | 13.5676 | zinc finger protein on ecdysone puffs |  |  |
|  | H3K27me3-only TSS | | | | |
|  | LOC105228715 | 3.37 | juvenile hormone acid O-methyltransferase/JHAMT | *JH* | Flatt et al., 2005; Jones et al., 2018; Wang et al., 2012 |
|  | LOC105232270 | 2.51 | Haemolymph juvenile hormone binding protein/JHBP |  |  |
|  | LOC105225924 | 3.77286 | ecdysone 20-monooxygenase | ecdysone | Lin et al., 2018 |
|  | LOC105227138 | 2.773335 | 20-hydroxyecdysone protein |  |  |
|  | H3K27me3-body | | | | |
|  | LOC105225924 | 3.170295 | ecdysone 20-monooxygenase | ecdysone | Lin et al., 2018 |
|  | LOC105227138 | 2.773335 | 20-hydroxyecdysone protein |  |  |
| 6.Octopamine synthesis | H3K4me3-only TSS | | | | |
|  | LOC105225586 | 23.5026 | probable G-protein coupled receptor No18 | *octopamine* (*OA*) | Roeder, 2020 |
|  | H3K27me3-only TSS | | | | |
|  | LOC105232537 | 2.470505 | octopamine receptor beta-3R | *OA* | Roeder, 2020 |
|  | LOC105232546 | 2.05729 | octopamine receptor beta-1R |  |  |
|  | H3K27me3-body | | | | |
|  | LOC105232537 | 2.470505 | octopamine receptor beta-3R | *OA* | Roeder, 2020 |
|  | LOC105232546 | 2.05729 | octopamine receptor beta-1R |  |  |
| 7.Neuropeptide hormones | H3K4me3-only TSS | | | | |
|  | LOC105226646 | 189.597 | peptide deformylase, mitochondrial | Neuropeptide | Nässel & Winther, 2010 |
|  | LOC105227755 | 22.413 | ion transport peptide |  |  |
|  | H3K27me3-only TSS | | | | |
|  | LOC105230385 | 2.532205 | Pigment-dispersing factor (PDF) receptor | Neuropeptide | Nässel & Winther, 2010 |
|  | LOC105226104 | 2.28473 | eclosion hormone |  |  |
|  | LOC105233453 | 2.3588 | RYamide receptor |  |  |
|  | LOC105228419 | 2.442305 | neuropeptide-like 1 |  |  |
|  | H3K27me3-body | | | | |
|  | LOC105226104 | 2.592385 | eclosion hormone | Neuropeptide | Nässel & Winther, 2010 |
|  | LOC105233453 | 2.6427 | RYamide receptor |  |  |
|  | LOC105228106 | 2.442305 | neuropeptide CCHamide-2 receptor |  |  |
|  | LOC105228419 | 2.3702 | neuropeptide-like 1 |  |  |
| 8.Muscle function | H3K4me3-only TSS | | | | |
|  | LOC105222384 | 4.02702 | laminin subunit beta-1 | lamins | Uchino et al., 2013 |
|  | LOC105227610 | 169.283 | phosphatidylserine decarboxylase proenzyme, mitochondrial |  |  |
|  | LOC105227979 | 155.462 | glycine cleavage system H protein, mitochondrial |  |  |
|  | LOC105229673 | 16.0864 | filamin-C |  |  |
|  | LOC105230056 | 8.97818 | laminin subunit gamma-1 |  |  |
|  | LOC105231194 | 183.855 | protein D3 |  |  |
|  | LOC105234056 | 133.381 | methylmalonic aciduria and homocystinuria type D homolog, mitochondrial |  |  |
|  | LOC105222016 | 125.384 | transmembrane protein KIAA1109 |  |  |
|  | LOC105227083 | 28.9534 | voltage-dependent calcium channel type D subunit alpha-1 |  |  |
|  | LOC105228118 | 43.5407 | lamin-C |  |  |
|  | LOC105223860 | 49.4093 | lamin-B receptor |  |  |
|  | LOC105232696 | 8.71601 | laminin subunit alpha |  |  |
|  | LOC105222038 | 233.769 | actin-related protein 2/3 complex subunit 4 | *Actin* | Hiromi & Hotta, 1985 |
|  | LOC105222053 | 108.288 | actin-related protein 2/3 complex subunit 2 |  |  |
|  | LOC105222184 | 14.5376 | thymosin beta |  |  |
|  | LOC105222336 | 188.44 | WASH complex subunit 1 |  |  |
|  | LOC105222768 | 36.0661 | actin, cytoplasmic A3a |  |  |
|  | LOC105223730 | 127.476 | actin-related protein 2/3 complex subunit 1A-B |  |  |
|  | LOC105224127 | 100.076 | beta-parvin |  |  |
|  | LOC105224276 | 115.54 | drebrin-like protein |  |  |
|  | LOC105224586 | 39.4676 | transmembrane protein 245 |  |  |
|  | LOC105225460 | 121.493 | protein flightless-1 |  |  |
|  | LOC105225552 | 64.2537 | moesin/ezrin/radixin homolog 1 |  |  |
|  | LOC105225618 | 68.6597 | alpha-actinin, sarcomeric |  |  |
|  | LOC105226160 | 219.228 | actin-related protein 6 |  |  |
|  | LOC105226770 | 51.9501 | tropomodulin |  |  |
|  | LOC105227714 | 192.072 | actin-like protein 6B |  |  |
|  | LOC105227798 | 90.3811 | F-actin-capping protein subunit beta |  |  |
|  | LOC105228161 | 68.5192 | cofilin/actin-depolymerizing factor homolog |  |  |
|  | LOC105228760 | 58.5809 | glia maturation factor beta |  |  |
|  | LOC105229330 | 104.166 | adenylyl cyclase-associated protein 1 |  |  |
|  | LOC105229580 | 10.6508 | actin, indirect flight muscle |  |  |
|  | LOC105229582 | 83.1309 | ataxin-2 homolog |  |  |
|  | LOC105229789 | 123.99 | actin-related protein 2 |  |  |
|  | LOC105230361 | 18.3822 | mucin-5AC |  |  |
|  | LOC105230630 | 103.971 | F-actin-capping protein subunit alpha |  |  |
|  | LOC105231057 | 59.5214 | twinfilin |  |  |
|  | LOC105231899 | 180.078 | dynactin subunit 4 |  |  |
|  | LOC105231987 | 133.299 | moesin/ezrin/radixin homolog 2 |  |  |
|  | LOC105232016 | 66.0069 | skin secretory protein xP2 |  |  |
|  | LOC105232679 | 31.6613 | huntingtin-interacting protein 1 |  |  |
|  | LOC105233223 | 100.782 | profilin |  |  |
|  | LOC105233791 | 141.089 | neural Wiskott-Aldrich syndrome protein |  |  |
|  | LOC105222303 | 152.408 | WAS/WASL-interacting protein family member 2 |  |  |
|  | LOC105223334 | 212.427 | protein asunder |  |  |
|  | LOC115066443 | 113.272 | protein furry-like |  |  |
|  | LOC105226992 | 47.0514 | titin | *sallimus* (*sls*) | Bullard et al., 2006 |
|  | LOC105230083 | 26.0681 | flightin | *flightin* | Reedy et al., 2000 |
|  | LOC105225609 | 173.587 | collagen type IV alpha-3-binding protein | collagen | Jones et al., 2015 |
|  | H3K27me3-only TSS | | | | |
|  | LOC105225125 | 2.637605 | villin-like protein quail | *Actin* | Hiromi & Hotta, 1985 |
|  | LOC105225453 | 3.272885 | actin-2, muscle-specific |  |  |
|  | LOC105229886 | 2.471885 | gelsolin |  |  |
|  | LOC105226720 | 2.81591 | collagen alpha chain CG42342 | collagen | Jones et al., 2015 |
|  | bivalent | | | | |
|  | LOC105230483 | 22.7298/2.9404 | basement membrane-specific heparan sulfate proteoglycan core protein | lamins | Uchino et al., 2013 |
|  | LOC105229245 | 14.4174/2.47048 | protein spire | Actin | Hiromi & Hotta, 1985 |
|  | LOC105225810 | 4.70182/3.99914 | collagen alpha chain CG42342 | collagen | Jones et al., 2015 |
|  | H3K27me3-body | | | | |
|  | LOC105222440 | 3.987935 | uncharacterized | lamins | Uchino et al., 2013 |
|  | LOC105228810 | 3.15232 | netrin-A |  |  |
|  | LOC105230056 | 2.9404 | laminin subunit gamma-1 |  |  |
|  | LOC105230483 | 3.127046667 | basement membrane-specific heparan sulfate proteoglycan core protein |  |  |
|  | LOC105231939 | 2.832506667 | filamin-A |  |  |
|  | LOC105232149 | 2.708485 | uncharacterized protein CG3556 |  |  |
|  | LOC105223499 | 2.32258 | protein cappuccino | *Actin* | Hiromi & Hotta, 1985 |
|  | LOC105225125 | 2.637605 | villin-like protein quail |  |  |
|  | LOC105225453 | 3.272885 | actin-2, muscle-specific |  |  |
|  | LOC105226770 | 2.47048 | tropomodulin |  |  |
|  | LOC105229245 | 3.239286667 | protein spire |  |  |
|  | LOC105229856 | 3.05876 | uncharacterized |  |  |
|  | LOC105229886 | 2.471885 | gelsolin |  |  |
|  | LOC105229934 | 2.768075 | protein Shroom |  |  |
|  | LOC105230361 | 2.30242 | mucin-5AC |  |  |
|  | LOC105233645 | 2.893365 | collagen alpha-1(V) chain | collagen | Jones et al., 2015 |
|  | LOC105225810 | 4.43616 | collagen alpha chain CG42342 |  |  |
| 9.Transforming growth factor β signalling | H3K4me3-only TSS | | | | |
|  | LOC105224809 | 14.5125 | uncharacterized | *bone morphogenetic proteins* (*BMPs*) | Doyle et al., 2022 |
|  | LOC105223255 | 46.5928 | uncharacterized |  |  |
|  | LOC105228764 | 83.1309 | bone morphogenetic protein receptor type-1B | *TGF-β* superfamily | Upadhyay et al., 2017 |
|  | LOC105224558 | 222.66 | TGF-beta receptor type-1 |  |  |
|  | H3K27me3-only TSS | | | | |
|  | LOC105231258 | 2.54831 | uncharacterized | *BMPs* | Doyle et al., 2022 |
|  | LOC105225826 | 2.429 | suppressor of lurcher protein 1 |  |  |
|  | LOC105232807 | 2.730925 | uncharacterized |  |  |
|  | LOC105225122 | 2.81635 | protein decapentaplegic | *TGF-β* superfamily | Upadhyay et al., 2017 |
|  | LOC105225678 | 3.10146 | inhibin beta A chain |  |  |
|  | H3K27me3-body | | | | |
|  | LOC105224809 | 2.43059 | uncharacterized | *BMPs* | Doyle et al., 2022 |
|  | LOC105223255 | 3.85689 | uncharacterized |  |  |
|  | LOC105231258 | 2.54831 | uncharacterized |  |  |
|  | LOC105221859 | 2.579605 | uncharacterized |  |  |
|  | LOC105225826 | 2.429 | suppressor of lurcher protein 1 |  |  |
|  | LOC105226800 | 5.698135 | cubilin |  |  |
|  | LOC105232807 | 2.730925 | uncharacterized |  |  |
| 10.JAK/STAT pathway and stress and immunity genes | H3K4me3-only TSS | | | | |
|  | LOC105226864 | 22.6749 | transcription factor Ken | *JAK/STAT* pathway | Doyle et al., 2022 |
|  | LOC105231837 | 14.2995 | signal transducer and transcription activator |  |  |
|  | LOC105231839 | 114.858 | signal transducer and transcription activator |  |  |
|  | LOC105231537 | 4.75 | cytochrome P450 6g1 | *cytochrome P450 monooxygenase* (*CYP450s*) | Jiao et al., 2020; Li et al., 2007; Scott & Wen, 2001 |
|  | LOC105233810 | 23.67 | probable cytochrome P450 6g2 |  |  |
|  | LOC105234048 | 25.39 | NADPH--cytochrome P450 reductase |  |  |
|  | LOC105224281 | 60.49 | probable cytochrome P450 305a1 |  |  |
|  | LOC105224875 | 19.39 | cytochrome P450 302a1, mitochondrial |  |  |
|  | LOC105225364 | 19.32 | cytochrome P450 9b2 |  |  |
|  | LOC105225363 | 14.29 | cytochrome P450 9b2 |  |  |
|  | LOC105225361 | 5.87 | probable cytochrome P450 9h1 |  |  |
|  | LOC105226034 | 3.51 | probable cytochrome P450 6d4 |  |  |
|  | LOC105228038 | 11.37 | probable cytochrome P450 6a14 |  |  |
|  | LOC105228051 | 5.89 | cytochrome P450 4e2 |  |  |
|  | LOC105230199 | 18.19 | probable cytochrome P450 317a1 |  |  |
|  | LOC105230212 | 54.29 | cytochrome P450 6a2 |  |  |
|  | LOC105230203 | 12.04 | cytochrome P450 6a9 |  |  |
|  | LOC105230949 | 10.94 | probable cytochrome P450 6a14 |  |  |
|  | LOC105232220 | 34.72 | cytochrome P450 4ae1 |  |  |
|  | LOC105224881 | 89.4524 | pyrimidodiazepine synthase-like | *Glutathione transferase* (*GST*) | Gonis et al., 2022 |
|  | LOC105224880 | 4.70046 | pyrimidodiazepine synthase |  |  |
|  | LOC105228364 | 102.638 | glutathione S-transferase theta-3 |  |  |
|  | LOC105229682 | 13.3826 | glutathione S-transferase 1 |  |  |
|  | LOC105225466 | 17.3023 | glutathione S-transferase 1 |  |  |
|  | LOC105225815 | 6.03676 | glutathione S-transferase 1-1-like |  |  |
|  | LOC105222104 | 14.2995 | glutathione S-transferase D1 |  |  |
|  | LOC105222427 | 5.42882 | glutathione S-transferase D7 |  |  |
|  | LOC105223110 | 89.2158 | glutathione S-transferase C-terminal domain-containing protein homolog |  |  |
|  | LOC105225812 | 8.87152 | glutathione S-transferase 1-1 |  |  |
|  | LOC105225813 | 15.306 | glutathione S-transferase D1 |  |  |
|  | LOC105225994 | 95.0173 | glutathione S-transferase 1 |  |  |
|  | LOC105228298 | 7.35459 | glutathione S-transferase theta-1 |  |  |
|  | LOC105228691 | 6.98403 | glutathione S-transferase S1 |  |  |
|  | LOC105225871 | 4.70046 | glutathione S-transferase 1-1 |  |  |
|  | LOC105222842 | 30.1629 | esterase B1 | *Esterase* | Montella et al., 2012 |
|  | LOC105224147 | 21.3103 | esterase FE4 |  |  |
|  | LOC105230315 | 28.9534 | esterase B1 |  |  |
|  | LOC105230316 | 84.6742 | esterase B1 |  |  |
|  | LOC105230317 | 32.5622 | esterase B1 |  |  |
|  | LOC105230320 | 17.8367 | esterase B1 |  |  |
|  | LOC105231880 | 142.33 | esterase GA18864 |  |  |
|  | LOC105222958 | 181.538 | KH domain-containing, RNA-binding, signal transduction-associated protein 3 | *multiple ankyrin repeats single KH domain* (*mask*) | Zhu et al., 2015 |
|  | LOC105228462 | 138.755 | KH domain-containing, RNA-binding, signal transduction-associated protein 2 |  |  |
|  | LOC105229281 | 90.0376 | tudor and KH domain-containing protein homolog |  |  |
|  | LOC105231560 | 125.131 | KH domain-containing, RNA-binding, signal transduction-associated protein 3 |  |  |
|  | LOC105231561 | 175.204 | KH domain-containing, RNA-binding, signal transduction-associated protein 2 |  |  |
|  | LOC105228459 | 87.0662 | KH domain-containing, RNA-binding, signal transduction-associated protein 3 |  |  |
|  | LOC105226358 | 93.0853 | ankyrin repeat and KH domain-containing protein mask |  |  |
|  | H3K27me3-only TSS | | | | |
|  | LOC105233910 | 3.37 | cytochrome P450 4c3 | *CYP450s* | Jiao et al., 2020; Li et al., 2007; Scott & Wen, 2001 |
|  | LOC105224924 | 2.88 | cytochrome P450 18a1 |  |  |
|  | LOC105228198 | 3.01 | cytochrome P450 6a9 |  |  |
|  | LOC105228765 | 6.13 | probable cytochrome P450 4ac1 |  |  |
|  | LOC105230198 | 2.63 | probable cytochrome P450 6a13 |  |  |
|  | LOC105230713 | 2.89 | probable cytochrome P450 301a1, mitochondrial |  |  |
|  | LOC105230837 | 2.92 | probable cytochrome P450 313a4 |  |  |
|  | LOC105231489 | 2.87 | probable cytochrome P450 316a1 |  |  |
|  | LOC105229690 | 5.11487 | glutathione S-transferase 1-like | *GST* | Gonis et al., 2022 |
|  | H3K27me3-body | | | | |
|  | LOC105227474 | 2.44773 | cytochrome P450 6a2 | *CYP450s* | Jiao et al., 2020; Li et al., 2007; Scott & Wen, 2001 |
|  | LOC105233910 | 2.76975 | cytochrome P450 4c3 |  |  |
|  | LOC105222002 | 2.76668 | cytochrome P450 315a1, mitochondrial-like |  |  |
|  | LOC105224923 | 2.560945 | cytochrome P450 306a1 |  |  |
|  | LOC105224924 | 2.87693 | cytochrome P450 18a1 |  |  |
|  | LOC105226935 | 3.23911 | probable cytochrome P450 6v1 |  |  |
|  | LOC105228198 | 2.37628 | cytochrome P450 6a9-like |  |  |
|  | LOC105228053 | 2.842275 | probable cytochrome P450 4ad1 |  |  |
|  | LOC105230198 | 2.634505 | probable cytochrome P450 6a13 |  |  |
|  | LOC105230713 | 2.88782 | probable cytochrome P450 301a1, mitochondrial |  |  |
|  | LOC105230837 | 3.947255 | probable cytochrome P450 313a4 |  |  |
|  | LOC105231489 | 2.87138 | probable cytochrome P450 316a1 |  |  |

**Table S3**. Summary of read mapping statistics.

| Sample | Total reads | | Specific mapped reads | | Non-specific reads | | Not mapped reads | |
| --- | --- | --- | --- | --- | --- | --- | --- | --- |
|  | Count | Percentage | Count | Percentage | Count | Percentage | Count | Percentage |
| Input | 39,099,728 | 100.00% | 14,587,568 | 37.31% | 1,330,314 | 3.40% | 23,181,846 | 59.29% |
| H3K4me3_1 | 35,807,534 | 100.00% | 19,820,420 | 55.35% | 801,722 | 2.24% | 15,185,392 | 42.41% |
| H3K4me3_2 | 38,248,934 | 100.00% | 21,295,174 | 55.68% | 843,790 | 2.21% | 16,109,970 | 42.12% |
| H3K27me3_1 | 45,894,890 | 100.00% | 18,453,022 | 40.21% | 1,198,680 | 2.61% | 26,243,188 | 57.18% |
| H3K27me3_2 | 41,296,776 | 100.00% | 16,625,576 | 40.26% | 989,856 | 2.40% | 23,681,344 | 57.34% |

**References**

Barnes, P. T., & Laurie-Ahlberg, C. C. (1986). Genetic variability of flight metabolism in *Drosophila melanogaster*. III. Effects of GPDH allozymes and environmental temperature on power output. *Genetics*, 112(2), 267-294.

Brisson, J. A., Ishikawa, A., & Miura, T. (2010). Wing development genes of the pea aphid and differential gene expression between winged and unwinged morphs. *Insect Molecular Biology, 19*, 63-73.

Bullard, B., Burkart, C., Labeit, S., & Leonard, K. (2005). The function of elastic proteins in the oscillatory contraction of insect flight muscle. *Journal of Muscle Research & Cell Motility*, 26(6), 479-485.

Clark-Hachtel, C. M., Linz, D. M., & Tomoyasu, Y. (2013). Insights into insect wing origin provided by functional analysis of vestigial in the red flour beetle, *Tribolium castaneum*. *Proceedings of the National Academy of Sciences, 110*(42), 16951-16956.

Colinet, H., Lee, S. F., & Hoffmann, A. (2010). Temporal expression of heat shock genes during cold stress and recovery from chill coma in adult Drosophila melanogaster. The FEBS journal, 277(1), 174-185.

Doyle, T., Jimenez‐Guri, E., Hawkes, W. L., Massy, R., Mantica, F., Permanyer, J., Cozzuto, L., Hermoso Pulido, T., Baril, T., & Hayward, A. (2022). Genome‐wide transcriptomic changes reveal the genetic pathways involved in insect migration. *Molecular ecology*, 31(16), 4332-4350.

Eanes, W. F., Merritt, T. J., Flowers, J. M., Kumagai, S., Sezgin, E., & Zhu, C.T. (2006). Flux control and excess capacity in the enzymes of glycolysis and their relationship to flight metabolism in *Drosophila melanogaster*. *Proceedings of the National Academy of Sciences*, 103(51), 19413-19418.

Flatt, T., Tu, M. P., & Tatar, M. (2005). Hormonal pleiotropy and the juvenile hormone regulation of *Drosophila* development and life history. *Bioessays, 27*(10), 999-1010.

Gonis, E., Fraichard, S., Chertemps, T., Hecker, A., Schwartz, M., Canon, F., & Neiers, F. (2022). Expression patterns of Drosophila Melanogaster glutathione transferases. *Insects*, 13(7), 612.

Gu, X., Zhao, Y., Su, Y., Wu, J., Wang, Z., Hu, J., Liu, L., Zhao, Z., Hoffmann, A. A., Chen, B., & Li, Z. (2019). A transcriptional and functional analysis of heat hardening in two invasive fruit fly species, Bactrocera dorsalis and Bactrocera correcta. Evolutionary Applications, 12(6), 1147-1163.

Hiromi, Y., & Hotta, Y. (1985). Actin gene mutations in *Drosophila*; heat shock activation in the indirect flight muscles. *The EMBO journal*, 4(7), 1681-1687.

Inomata, N., Takahasi, K. R., & Koga, N. (2019). Association between duplicated maltase genes and the transcriptional regulation for the carbohydrate changes in *Drosophila melanogaster*. *Gene*, 686, 141-145.

Jeffries, K. A., Dempsey, D. R., Behari, A. L., Anderson, R. L., & Merkler, D. J. (2014). *Drosophila melanogaster* as a model system to study long-chain fatty acid amide metabolism. *FEBS letters*, 588(9), 1596-1602.

Jiao, L., Zhang, X.Y., Wu, H.H., Wen, M., Zhu, W.Y., Kun-Yan, Z., & Zhang, J.Z. (2020). Characteristics and roles of cytochrome b5 in cytochrome P450-mediated oxidative reactions in Locusta migratoria. Journal of Integrative Agriculture, 19(6), 1512-1521.

Jones, C. M., Lim, K. S., Chapman, J. W., & Bass, C. (2018). Genome-wide characterization of DNA methylation in an invasive lepidopteran pest, the cotton bollworm *Helicoverpa armigera*. *G3: Genes, Genomes, Genetics, 8*(3), 779-787.

Jones, C. M., Papanicolaou, A., Mironidis, G. K., Vontas, J., Yang, Y., Lim, K. S., Oakeshott, J. G., Bass, C., & Chapman, J. W. (2015). Genomewide transcriptional signatures of migratory flight activity in a globally invasive insect pest. *Molecular ecology*, 24(19), 4901-4911.

Kanamori, Y., Saito, A., Hagiwara-Komoda, Y., Tanaka, D., Mitsumasu, K., Kikuta, S., Watanabe, M., Cornette, R., Kikawada, T., & Okuda, T. (2010). The trehalose transporter 1 gene sequence is conserved in insects and encodes proteins with different kinetic properties involved in trehalose import into peripheral tissues. *Insect biochemistry and molecular biology*, 40(1), 30-37.

Li, F.F., Jun, X., Li, J.M., Liu, S.S., & WANG, X.W. (2012). p38 MAPK is a component of the signal transduction pathway triggering cold stress response in the MED cryptic species of *Bemisia tabaci*. *Journal of Integrative Agriculture*, 11(2), 303-311.

Li, X., Schuler, M. A., & Berenbaum, M. R. (2007). Molecular mechanisms of metabolic resistance to synthetic and natural xenobiotics. Annual Review Entomology, 52, 231-253.

Lin, X., & Lavine, L. C. (2018). Endocrine regulation of a dispersal polymorphism in winged insects: a short review. *Current opinion in insect science*, 25, 20-24.

Lindquist, S. (1986). The heat-shock response. Annual review of biochemistry, 55(1), 1151-1191.

Liu, F., Li, X., Zhao, M., Guo, M., Han, K., Dong, X., Zhao, J., Cai, W., Zhang, Q., & Hua, H. (2020). Ultrabithorax is a key regulator for the dimorphism of wings, a main cause for the outbreak of planthoppers in rice. *National Science Review, 7*(7), 1181-1189.

Mattila, J., & Hietakangas, V. (2017). Regulation of carbohydrate energy metabolism in *Drosophila melanogaster*. *Genetics, 207*(4), 1231-1253.

Montella, I. R., Schama, R., & Valle, D. (2012). The classification of esterases: an important gene family involved in insecticide resistance-A review. *Memorias do Instituto Oswaldo Cruz*, 107, 437-449.

Nässel, D. R., Kubrak, O. A., Liu, Y., Luo, J., & Lushchak, O. V. (2013). Factors that regulate insulin producing cells and their output in *Drosophila*. *Frontiers in Physiology, 4*, 252.

Nässel, D. R., & Winther, Å. M. (2010). *Drosophila neuropeptides* in regulation of physiology and behavior. *Progress in neurobiology*, 92(1), 42-104.

Niwa, N., Akimoto‐Kato, A., Niimi, T., Tojo, K., Machida, R., & Hayashi, S. (2010). Evolutionary origin of the insect wing via integration of two developmental modules. *Evolution & Development, 12*(2), 168-176.

O’Donnell, B. C., & Jockusch, E. L. (2010). The expression of wingless and Engrailed in developing embryos of the mayfly *Ephoron leukon* (Ephemeroptera: Polymitarcyidae). *Development Genes and Evolution, 220*(1), 11-24.

Parisi, F., Riccardo, S., Zola, S., Lora, C., Grifoni, D., Brown, L. M., & Bellosta, P. (2013). dMyc expression in the fat body affects DILP2 release and increases the expression of the fat desaturase Desat1 resulting in organismal growth. *Developmental biology*, 379(1), 64-75.

Paul, L., Wang, S.-H., Manivannan, S. N., Bonanno, L., Lewis, S., Austin, C. L., & Simcox, A. (2013). Dpp-induced Egfr signaling triggers postembryonic wing development in *Drosophila*. *Proceedings of the National Academy of Sciences, 110*(13), 5058-5063.

Quah, S., Hui, J. H., & Holland, P. W. (2015). A burst of miRNA innovation in the early evolution of butterflies and moths. *Molecular Biology and Evolution, 32*(5), 1161-1174.

Reedy, M. C., Bullard, B., & Vigoreaux, J. O. (2000). Flightin is essential for thick filament assembly and sarcomere stability in *Drosophila* flight muscles. *The Journal of cell biology*, 151(7), 1483-1500.

Roch, F., Jiménez, G., & Casanova, J. (2002). EGFR signalling inhibits Capicua-dependent repression during specification of *Drosophila* wing veins. *Development*, *129*(4): 993-1002.

Roeder, T. (2020). The control of metabolic traits by octopamine and tyramine in invertebrates. *Journal of Experimental Biology*, 223(7), jeb194282.

Rose, J. B., Crews, L., Rockenstein, E., Adame, A., Mante, M., Hersh, L. B., Gage, F. H., Spencer, B., Potkar, R., & Marr, R. A. (2009). Neuropeptide Y fragments derived from neprilysin processing are neuroprotective in a transgenic model of Alzheimer's disease. *Journal of Neuroscience*, 29(4), 1115-1125.

Scott, J. G., & Wen, Z. (2001). Cytochromes P450 of insects: the tip of the iceberg. Pest management science, 57(10), 958-967.

Shen, J., Dahmann, C., & Pflugfelder, G. O. (2010). Spatial discontinuity of optomotor-blind expression in the *Drosophila* wing imaginal disc disrupts epithelial architecture and promotes cell sorting. *BMC Developmental Biology, 10*(1), 1-12.

Strigini, M., & Cohen, S. M. (2000). Wingless gradient formation in the *Drosophila* wing. *Current Biology, 10*(6), 293-300.

Tomoyasu, Y. (2017). Ultrabithorax and the evolution of insect forewing/hindwing differentiation. *Current Opinion in Insect Science, 19*, 8-15.

Tomoyasu, Y., Ohde, T., & Clark-Hachtel, C. (2017). What serial homologs can tell us about the origin of insect wings. *F1000Research, 6*.

Trempolec, N., Dave-Coll, N., & Nebreda, A. R. (2013). SnapShot: p38 MAPK signaling. *Cell*, 152(3), 656-656. e651.

Turrel, O., Lampin-Saint-Amaux, A., Préat, T., & Goguel, V. (2016). *Drosophila* neprilysins are involved in middle-term and long-term memory. *Journal of Neuroscience*, 36(37), 9535-9546.

Uchino, R., Nonaka, Y.-k., Horigome, T., Sugiyama, S., & Furukawa, K. (2013). Loss of *Drosophila* A-type lamin C initially causes tendon abnormality including disintegration of cytoskeleton and nuclear lamina in muscular defects. *Developmental biology*, 373(1), 216-227.

Upadhyay, A., Moss-Taylor, L., Kim, M.J., Ghosh, A. C., & O’Connor, M. B. (2017). Erratum: TGF-β family signaling in *Drosophila*. *Cold Spring Harbor perspectives in biology*, 9(3).

Wang, S., Minter, M., Homem, R. A., Michaelson, L. V., Venthur, H., Lim, K. S., Withers, A., Xi, J., Jones, C. M., & Zhou, J. J. (2020). Odorant binding proteins promote flight activity in the migratory insect, *Helicoverpa armigera*. *Molecular ecology*, 29(19), 3795-3808.

Wang, Y., Brent, C. S., Fennern, E., & Amdam, G. V. (2012). Gustatory perception and fat body energy metabolism are jointly affected by vitellogenin and juvenile hormone in honey bees. *PLoS Genetics, 8*(6), e1002779.

Zecca, M., & Struhl, G. (2002). Subdivision of the *Drosophila* wing imaginal disc by EGFR-mediated signaling. *Development*, *129*(6): 1357-1368.

Zhu, M., Li, X., Tian, X., & Wu, C. (2015). Mask loss-of-function rescues mitochondrial impairment and muscle degeneration of *Drosophila* pink1 and parkin mutants. *Human molecular genetics*, 24(11), 3272-3285.

Table S4-S7 are provided separately as excel spreadsheets in **Supplementary File S2.**

**Table S4**. List and location of H3K4me3 and H3K27me3 peaks in the *Bactrocera dorsalis* genome.

**Table S5**. List and annotation of the genes with H3K4me3-only, H3K27me3-only and bivalent domains at the TSSs, and genes with H3K27me3 at the gene body regions in the *Bactrocera dorsalis* genome.

**Table S6**. List with all over-represented GO terms of genes associated with Cluster 1-3 and genes with H3K4me3-only, H3K27me3-only and bivalent domains at the TSSs, and genes with H3K27me3 at the gene body regions in the *Bactrocera dorsalis* genome.

**Table S7**. Summary of the sequences containing putative H3K4me3, H3K27me3 and bivalent TSSs motifs and the motif position information for genes related to insect flight activity in *Bactrocera dorsalis*.

**Supplementary File S3.**

ChIP-seq: Part 1 showed the code for trimming, filtering and mapping reads, Part 2 showed the code for peak calling, annotation, and visualization, Part 3 the code for showed GO analysis, and Part 4 showed the code for Motif analysis.

RNA-seq: Part 1 showed the code for trimming, filtering and mapping reads, Part 2 showed the code for quantifying the reads count.
